# Effects of the level and source of dietary physically effective fiber on feed intake, nutrient utilization, heat energy, ruminal fermentation, and milk production by Alpine goats

**DOI:** 10.1016/j.aninu.2024.02.002

**Published:** 2024-03-04

**Authors:** Raquel V. Lourencon, Amlan K. Patra, Luana P.S. Ribeiro, Ryszard Puchala, Wei Wang, Terry A. Gipson, Arthur L. Goetsch

**Affiliations:** American Institute for Goat Research, Langston University, Langston, OK, USA

**Keywords:** Physically effective fiber, Alpine goat, Lactation, Ruminal fermentation, Feeding behavior

## Abstract

Thirty-two primiparous and 31 multiparous Alpine goats were used to determine influences of diets varying in level and source of forage on performance in early to mid-lactation for 16 wk. Diets consisted of 40%, 50%, 60%, and 70% forage (designated as 40F, 50F, 60F, and 70F, respectively) with 60F and 70F containing coarsely ground grass hay (primarily orchardgrass) and 40F and 50F containing cottonseed hulls, alfalfa pellets, and coarsely ground wheat hay. Diets contained 15.9% to 16.3% crude protein and 37.8%, 42.1%, 53.5%, and 55.4% neutral detergent fiber (NDF) with 10.0%, 15.8%, 50.1%, and 55.5% particle retention on a 19-mm sieve, and 26.1%, 29.6%, 38.3%, and 40.0% physically effective NDF (peNDF) for 40F, 50F, 60F, and 70F, respectively. Dry matter intake (2.71, 2.75, 1.96, and 1.95 kg/d) and milk yield (2.82, 2.71, 2.23, and 2.10 kg/d for 40F, 50F, 60F, and 70F, respectively) were lower (*P* < 0.05) for the two diets highest in forage. Digestion of organic matter was similar among diets (*P >* 0.05), but digestibility of NDF was greater (*P* < 0.05) for 60F and 70F (57.5%, 58.4%, 68.9%, and 72.2% for 40F, 50F, 60F, and 70F, respectively). Diet affected (*P* < 0.05) milk fat (3.16%, 3.37%, 2.93%, and 2.97%) and protein concentrations (2.62%, 2.69%, 2.58%, and 2.52% for 40F, 50F, 60F, and 70F, respectively). Milk energy yield was greater (*P* < 0.05) for the two diets lowest in forage (7.51, 7.45, 5.68, and 5.34 MJ/d), although yield relative to dry matter intake was not affected (*P* > 0.05) by diet and was lower (*P* < 0.05) for primiparous vs. multiparous goats (2.71 and 3.09 MJ/kg). Ruminal pH and acetate proportion were greater for 60F and 70F than for the other diets and the proportion of butyrate was lower for the two diets highest in fiber. The mean lengths of time spent ruminating, eating, standing, and lying were not affected (*P* > 0.05) by diet or parity, but many interactions involving diet, period, hour, and parity were significant (*P* < 0.05). In conclusion, lactational performance of Alpine goats in early to mid-lactation will be constrained with diets high in forage of moderate quality, peNDF content, and large particle size, which appeared related to limited feed intake.

## 1 Introduction

Feed accounts for a major portion of the cost of production in lactating dairy goats, and utilization of an optimum proportion of native forages can reduce diet cost compared with the use of concentrate feeds (Morand-Fehr, 2005). Although the concentration of nutrients and available energy are normally greater for concentrates than for feedstuffs higher in fiber, both types of feeds typically are included in diets depending on characteristics of the animal such as parity, stage of lactation, production potential and target, and price of specific feed ingredients. In regard to the latter factor, a relatively high level of home-grown feedstuffs might in some cases be selected due to their lower cost, even if it results in lower production levels compared with more expensive purchased ingredients. As a result, there is a trend to include a greater proportion of forages in total diet without compromising milk production. For instance, in regions of the USA such as the Northeast where high-quality forages can be grown consistently, many diets of dairy cattle are much higher in forage than previously and at the same time, milk production has increased due to inclusion of higher quality forages (Chase and Grant, 2013). Moreover, high-forage diets offer advantages such as less or no need for a ruminal buffering agent and lower risk of digestive upsets and other adverse health conditions.

Forage quality is an important factor determining feed intake and, thus, level of production by ruminants. Feed intake generally decreases with increasing amounts of forage and fiber in diets due to physical limitation of gut fill (Galyean and Owens, 1991; Giger-Reverdin et al., 2014; Serment et al., 2011), although this depends on many factors such as forage particle size and quality, physiological stages of animals, forage digestion, and passage rate. Although effective neutral detergent fiber (NDF), sometimes referred to as physically effective NDF (peNDF), is the function of particle size and chemical NDF concentration in forages, forage peNDF characteristics also depend upon other factors including particle shape, tensile strength, and degradation rate (Zhao et al., 2011). These factors ultimately determine feed intake, feeding behaviors, ruminal morphology, rumen volatile fatty acid (VFA) production, and microbial protein synthesis (Cao et al., 2021; Xue et al., 2022). The forage peNDF characteristics thus vary among different sources of forages that could impact performance of goats including milk production (Xue et al., 2022).

Because dairy goats may not have been as intensively selected for milk production as dairy cattle and exhibit higher digesta passage rate through the gastrointestinal tract and feed intake relative to body weight (BW) (Goetsch, 2016), perhaps there also is an opportunity for use of high levels of good quality forages without any or marked sacrifice of performance. Relatedly, some research has indicated less performance benefits from high dietary concentrate levels for dairy goats compared with dairy cattle (Goetsch et al., 2001; Morand-Fehr and Sauvant, 1978). Also, high dietary levels of fiber potentially thoroughly digested in the rumen could enhance microbial growth and ruminal acetate absorption (Cao et al., 2021; Robinson and McQueen, 1992), which would be expected to lead to increases in milk fat concentration and yield (Seymour et al., 2005; Urrutia et al., 2019). This is particularly relevant for breeds such as Alpine goats that can produce large quantities of milk but often with relatively low levels of fat as well as protein (Charpentier et al., 2019; Tovar-Luna et al., 2010). Furthermore, in a literature review Goetsch (2019) postulated that common practices of inclusion of low levels of low-quality fibrous roughages and the legume alfalfa in diets of high-yielding dairy goats could result in a more ‘grain-’ than ‘forage-like’ array of digestion end products conductive to low milk fat and protein concentrations. Based on the above considerations, it was hypothesized that the concentrations and sources of forages would influence performance and associated variables in lactating Alpine goats. Therefore, the objectives of this experiment were to determine effects of both different levels and sources of high-fiber feedstuffs differing in peNDF contents on performance, nutrient utilization, ruminal fermentation, and feeding behaviors in Alpine dairy goats.

## 2 Materials and methods

### 2.1 Animal ethics statement

The study was conducted according to the guidelines of the AWA and PHS policy and approved by the Langston University Animal Care and Use Committee (Approval Number: 19-10; 10 April 2019). The experimental design was compatible with the ARRIVE (Animal Research: Reporting of In Vivo Experiments) guidelines 2.0.

### 2.2 Animals, facility, and treatments

Thirty-two Alpine doelings in the first lactation (primiparous and parity of 1) and 32 does (multiparous and parity of 2 or greater) were allocated to 8 groups, with 4 animals of each parity, based on BW and age. Groups were randomly assigned to 4 treatments and each treatment had 15 or 16 animals. One doe developed a health issue early in the experiment and was removed. Initial BW and age of the primiparous goats were 56.1 ± 1.22 kg and 2.3 ± 0.08 years and of does were 58.3 ± 1.33 kg and 3.7 ± 0.24 years, respectively. A larger number of animals had been bred with estrus synchronization, with the experiment beginning at 13.8 ± 0.19 d-in-milk. The length of the performance monitoring period of the experiment was 16 wk, divided into four 4-wk periods. Thereafter, the animals continued on the treatments for an additional 1 to 2 wk to determine the ratio of heat energy (HE) to heart rate (HR) and to estimate digestibility.

The confinement facility used during the 16-wk experimental period, described by Patra et al. (2009), had 8 pens (5.57 m × 3.06 m). An area of 5.57 m × 1.33 m at the front of each pen had an elevated expanded metal floor, with a flush manure system used once daily. There were 4 pens (total 8 pens with 8 animals in 7 pens and 7 animals in 1 pen) on each side of the facility separated by a hallway adjacent to the feeders. One animal group of each treatment was on the same side, with a random allocation of groups to the sides. Ambient temperature and relative humidity were determined every 30 min with two Hobo Temperature/RH Data Loggers (model number U12-011; Onset Computer Corp., Bourne, MA, USA) placed in different areas of the facility.

The diets varied in both levels of concentrate or forage as well as sources of forage. Concentrate levels were 60%, 50%, 40%, and 30% and forage levels were 40%, 50%, 60%, and 70%, designated as 40F, 50F, 60F, and 70F, respectively (Table 1). The diets were prepared using Excel to fulfil the nutrient requirements for milk production of 3 to 5 L/d (NRC, 2007). The sole forage source in 60F and 70F diets was grass hay, primarily of orchardgrass (*Dactylis glomerta*). Conversely, 40F and 50F included a source of wheat hay (*Triticum aestivum*), dehydrated alfalfa pellets, and cottonseed hulls. The diets 40F to 70F were intended to create different forage levels, whereas 40F versus 50F and 60F versus 70F were considered to compare forage levels within sources. Simultaneously, two forage sources differing in quality may be compared though there were confounding factors. Changes in forage levels and sources resulted in differences in peNDF contents and forage particle sizes in diets. Before the study, samples of the two sources of grass hay were analyzed at different commercial laboratories. Based on those results and values for other feedstuffs of Preston (2015) and ingredient manufacturers, levels of dietary ingredients were varied to achieve target levels of various fractions. Diets were sampled daily to form weekly composite samples and were offered free-choice at 110% to 120% of consumption on the preceding few days in Calan gate feeders (American Calan Inc., Northwood, NH, USA) at 08:00.

**Table 1:** Composition of different diets with various levels and sources of forages fed to lactating Alpine goats.

Item	Diet^1^			
	40F	50F	60F	70F
Ingredient composition, % DM				
Dehydrated alfalfa pellets	7.50	12.50	0.00	0.00
Cottonseed hulls	25.00	25.00	0.00	0.00
Orchardgrass hay (coarsely ground)	0.00	0.00	60.00	70.00
Wheat hay (coarsely ground)	7.50	12.50	0.00	0.00
Wheat middlings	11.69	8.57	7.24	4.09
Rolled oats	11.68	8.57	7.24	4.09
Rolled corn	11.59	8.57	7.24	4.09
Soybean meal	14.84	14.44	1.25	0.21
Soyplus^2^	0.00	0.00	7.63	8.00
Megalac^3^	3.00	3.00	3.00	3.00
Molasses (liquid)	5.00	5.00	5.00	5.00
Dicalcium phosphate	0.19	0.37	0.00	0.14
Limestone	0.55	0.15	0.76	0.62
Sodium bicarbonate	0.60	0.50	0.00	0.00
Ammonium sulfate	0.10	0.10	0.15	0.14
Magnesium oxide	0.27	0.24	0.00	0.00
Ammonium chloride	0.00	0.00	0.00	0.13
Sodium chloride	0.40	0.40	0.40	0.40
Vitamin premix^4^	0.05	0.05	0.05	0.05
Trace mineral mix^5^	0.04	0.04	0.04	0.04
Chemical composition^6^				
Moisture, % fresh basis	11.5 ± 0.32	11.5 ± 0.51	10.7 ± 0.62	11.4 ± 2.32
Ash, % DM	9.7 ± 0.28	9.6 ± 0.13	9.1 ± 0.17	8.6 ± 0.17
Crude protein, % DM	15.9 ± 0.12	16.3 ± 0.29	16.3 ± 0.33	16.0 ± 0.27
Neutral detergent fiber, % DM	37.8 ± 0.50	42.1 ± 0.79	53.5 ± 1.19	55.4 ± 1.08
Acid detergent fiber, % DM	26.9 ± 0.46	30.5 ± 0.72	31.2 ± 0.91	32.2 ± 0.58
Acid detergent lignin, % DM	5.6 ± 0.12	6.7 ± 0.18	4.5 ± 0.23	4.8 ± 0.22
Total digestible nutrient, %^7^	69.9	67.8	70.6	68.1

DM = dry matter.

1 Diets were 40%, 50%, 60%, and 70% forage (40F, 50F, 60F, and 70F, respectively), with forage in 60F and 70F diet being grass hay (primarily orchardgrass) and that in 40F and 50F cottonseed hulls, dehydrated alfalfa pellets, and wheat hay.

2 Dairy Nutrition Plus, Ralston, IA, USA.

3 Church & Dwight, Ewing, NJ, USA.

4 Vitamin premix contained (per kilogram of diet): 8,800,000 IU vitamin A, 1,760,000 IU vitamin D_3_, and 1100 IU vitamin E.

5 Trace mineral mix contained (per kilogram of diet): 275 mg Co, 2000 mg I, 43,746 mg Fe, 750 mg Se, 18,748 mg Cu, 68,744 mg Zn, and 19,998 mg Mn.

6 Mean ± SEM based on 16 weekly samples.

7 Calculated values (Preston, 2015).

### 2.3 Measures

#### 2.3.1 Milk yield and composition

All does were milked every day at 07:00 and 17:00 in automatic milking machines (Bou-Matic, DEC International, Madison, WI, USA) and milk yield of each doe was recorded in a computerized system (Westfalia Systemat, Elk Grove Village, IL, USA). Milk samples were collected every 2 wk in the morning and afternoon and analyzed for fat, protein, lactose, total solids, solids-non-fat, and urea nitrogen (N) at the certified Dairy Herd Information Laboratory for Goats of Langston University with a DairySpec and use of NexGen v2.15+ software (Bentley Instruments Inc., Chaska, MN, USA). Somatic cell count (SCC) was determined with a Bentley SomaCount instrument. Milk energy concentration was determined with the equation of NRC (2001) based on concentrations of fat, protein, and lactose. Milk energy also was expressed relative to dry matter (DM) intake.

#### 2.3.2 Body weight, body condition score (BCS), and body mass indexes (BMI)

Body condition score assessed by three individuals as described by Ngwa et al. (2007) and BW using a portable digital weighing balance were determined at the beginning of the experiment and end of the periods. Linear measures were manually determined at the beginning, middle, and end of the experiment, designated in tabular form as periods 1, 2, and 4, respectively. Measures were height at withers (Wither), length from the point of the shoulder to hook bone (Hook) and pin bone (Pin), circumference from heart girth, and width at hook bones (Rump). Four of the 13 BMI were calculated as described by Liu et al. (2019) with BW in gram and linear measures in centimeter and noted below.

BMI-WH (g/cm^2^) = BW/(Wither × Hook);

BMI-WP (g/cm^2^) = BW/(Wither × Pin);

BMI-GH (g/cm^2^) = BW/(Heart girth × Hook);

BMI-GP (g/cm^2^) = BW/(Heart girth × Pin).

#### 2.3.3 Feed intake, digestibility, and Heat energy

Feed refusals were weighed before feeding each morning to determine feed intake. As noted earlier, after the 16-wk experiment, animals were transferred to another facility and situated in 0.7 m × 1.2 m metabolism crates. A portion of the animals were moved in the first week and this occurred for others in the second week. Total feces was collected for 5 d, and a composite sample was formed from 10% daily aliquots. Digestibility was based on feed intake, fecal excretion, and feed and feces composition during those weeks. Body weight was determined at the beginning and end of excreta collection days as well as the day of calorimetry measures.

Partial DM concentration in feces was determined by drying in a forced-air oven at 55 °C for 48 h. Feed and fecal samples were analyzed at a commercial laboratory (Custom Laboratory, Monett, MO, USA; https://www.customaglabs.com). The DM concentration was determined by a modified malt gravimetric method of AOAC (1990; no. 935.29), which entailed drying at 105 °C for 3 h. Levels of ash and Kjeldahl N were also determined by AOAC (1990) procedures (no. 942.05 and 988.05, respectively), with crude protein (CP) calculated as N × 6.25. An ANKOM fiber analyzer (ANKOM Technology, Macedon, NY, USA) was used to determine concentrations of NDF, acid detergent fiber (ADF), and acid detergent lignin (ADL) via procedures of ANKOM (https://www.ankom.product-catalog/ankom-200-fiber-analyzer; methods 6, 5, and 8, respectively), with modifications of Van Soest et al. (1991). The estimation of NDF included use of heat stable amylase and sodium sulfate. Levels of NDF and ADF included residual ash, whereas that of ADL did not. Particle size of feed samples was characterized with the Penn State Particle Separator (https://extension.psu.edu/penn-state-particle-separator). The content of peNDF, was calculated by multiplying the quantity of particles retained on the three sieves by diet DM and NDF concentrations.

During the 2 wk after the 16-wk experiment, animals were cycled in groups of up to seven into a room with metabolism crates fitted with headboxes of a respiration calorimetry system for 1 d to determine the ratio of HE to HR. Emission of methane and carbon dioxide and oxygen consumption were measured with an indirect, open-circuit respiration calorimetry system (Sable Systems International, North Las Vegas, NV, USA) as described by Puchala et al. (2007, 2009). Oxygen concentration was analyzed using a fuel cell FC-1B oxygen analyzer (Sable Systems International) and methane and carbon dioxide concentrations were measured with infrared analyzers (CA-1B for carbon dioxide and MA-1 for methane; Sable Systems International). Prior to gas exchange measurements, analyzers were calibrated with gases of known concentrations. Ethanol combustion tests were performed to ensure complete recovery of oxygen and carbon dioxide produced with the same flow rates as used during measurements. Heat energy was calculated from oxygen consumption and production of carbon dioxide and methane according to the Brouwer (1965) equation without consideration of urinary N.

For measurement of HR in the last week of each 4-wk period, animals were fitted with 10 cm × 10 cm electrodes prepared from stretch conductive fabric (Less EMF, Albany, NY, USA), glued to Vermed PerformancePlus ECG electrodes (Bellows Falls, VT, USA) and attached to the chest just behind and slightly below the left elbow and behind the shoulder blade on the right side. Electrodes were connected by ECG snap leads (Bioconnect, San Diego, CA, USA) to T61 coded transmitters (Polar, Lake Success, NY, USA). Human S610 heart rate (Polar) monitors with wireless connection to the transmitters were used to collect HR data at a 1-min interval. Heart rate data were analyzed using Polar Precision Performance SW software. Heat energy was calculated by application of the ratio of HE to HR determined with the respiration calorimetry system.

#### 2.3.4 Behavior

On the days of HR monitoring in periods 2 and 4, behavior also was characterized by use of two Hobo Pendant G loggers (Onset Computer Corp.) that measure acceleration and angular displacement in three (X, Y, and Z) axes to record the *g*-force. One logger was situated in a pouch of a halter so that it was positioned under the chin to assess side-to-side (X axis), back-and-forth (Y axis), and up-and-down (Z axis) movements at 10 s intervals. Halters were constructed using an elastic tape for the noseband, a spandex pouch for the logger, and adjustable velcro straps that fastened behind the head of the goats. The equations were further validated in a further study via video recording using cameras with built-in infrared capabilities (Canon VC-C50i Camera, Canon U.S.A., Inc., Melville, NY) and infrared spotlights. In each of the four periods, a second logger was held in a pouch situated on the inside of the left hind leg to record *g*-force and tilt on X-, Y-, and Z-axes at 1-min intervals (Zobel et al., 2015). The large, rounded end of the loggers oriented ventrally, and the medial rib of the logger oriented toward the leg. Data from loggers were downloaded via HoboWare Pro V3 (Onset Computer Corporation) and raw *g*-force values were exported, which were then imported into SAS (SAS Institute Inc., Cary, NC) for further analyses. Data from loggers do not include direct estimates of time ruminating or eating. Thus, equations to predict ruminating or eating was developed in a calibration study with classification tree analysis (Steinberg and Colla, 1997) and CART software (SPM Ver. 8, Salford Systems, San Diego, CA) analysis. Prediction equations for goats previously established (Ribeiro et al., 2019) were used to determine time spent ruminating, eating, and idle. Again, data from loggers do not include direct estimates of time lying or standing. A SAS program with the prediction equations supplied by Zobel et al. (2015) for goats were used to determine time spent standing and lying on the right and left sides.

#### 2.3.5 Ruminal fluid characteristics and blood constituents

Ruminal fluid was sampled by stomach tube in wk 3 of each period at 3 to 4 h after feeding on the first day, and before the afternoon milking at 7 to 8 h after feeding on the subsequent day. Ruminal fluid pH was measured with a digital meter, and a 3-mL sample was placed in a tube with 2 mL of 3 M HCl for ammonia N analysis by the procedure of Broderick and Kang (1980). An aliquot of 4 mL was dispensed into a tube with 1 mL of 25% (wt/vol) metaphosphoric acid for VFA analysis (Cottyn and Boucque, 1968) by gas chromatography (Agilent Technologies 6890N, Santa Clara, CA, USA) fitted with a capillary column (Agilent J&W DB-FFAP; 30 m length, 320 μm inner diameter and 0.25 μm film thickness) and a flame ionization detector as per following conditions: 1 μL injection volume; injector temperature of 250 °C; flow rates of 40, 450 and 30 mL/min for hydrogen, air, and nitrogen, respectively; initial oven temperature of 100 °C holding for 5 min, then raised at 10 °C/min to 125 °C, holding for 3 min, and detector temperature of 250 °C.

Blood was sampled by jugular venipuncture into three tubes at same times when ruminal fluid was collected. There were two tubes used for plasma, one with sodium fluoride and potassium oxalate and another sodium heparin. A third tube without an anticoagulant was used to derive serum. Plasma and serum were harvested by centrifugation for 20 min at 3000 × *g* and frozen at −20 °C. Plasma from the sodium fluoride and potassium oxalate tube was analyzed for glucose and lactate, respectively, with a USI 2300 Plus Glucose & Lactate Analyzer (YSI Inc., Yellow Springs, OH, USA). Serum was analyzed for total protein, albumin, triglycerides, and cholesterol with a Vet Axcel Chemistry Analyzer (Alfa Wassermann Diagnostic Technologies, West Caldwell, NJ, USA) to roughly understand the protein and energy status of animals.

### 2.4 Statistical analyses

Most data were analyzed by mixed effects models with the Statistical Analysis System (Littell et al., 1998; SAS, 2011). Somatic cell count was log-transformed for analysis. Variables with one value per period were analyzed with the following model:

*Y*_*ijkl*_ = *μ* + Diet_*i*_ + Parity_*j*_ + Period_*k*_ + Diet_*i*_ × Parity_*j*_ + Diet_*i*_ × Period_*k*_ + Parity_*j*_ × Period_*k*_ + Diet_*i*_ × Parity_*j*_ × Period_*k*_ + Error_*ijkl*_,

where, *Y*_*ijkl*_ is the observation from animal *l* in diet *i*, parity *j*, and period *k*; *μ* is the overall mean; Error_*ijkl*_ is the random residual error; Dietary treatment (*i* = 1–4), parity (*j* = 1–2), period (*k* = 1–4) as a repeated measure, and all interactions were fixed effects and animal within treatment × parity was random and the repeated measure subject. Variables associated with the digestibility trial were analyzed without Period in the model. For ruminal fluid and blood measures, time of sampling was included as an additional repeated measure. For most ruminal fluid variables, there were many interactions involving parity; therefore, the analyses were conducted by parity. Also, because of a significant three-way interaction in concentrations of isovalerate and butyrate in ruminal fluid of does, those analyses were conducted by period. The analysis for hourly values for behavior variables was similar, with hour of the day included as a repeated measure. Analyses were conducted by factors such as parity to address significant interactions.

Interaction means are presented when the interaction was significant (*P* < 0.05). In addition, because main effect means for some variables (e.g., milk yield) are of interest even with one or more significant interactions involving period, both main effect and interaction means are presented. The model for particle size measures included dietary treatment alone. Means were separated by least significant difference with a protected F-test. Because of significant interactions involving treatment, period, and(or) parity, these effects are primarily presented through supplementary tables, whereas main effects mainly addressed in the body of the article.

## 3 Results and discussion

### 3.1 Diets and environmental conditions

Levels of many feedstuffs were varied to achieve diet composition targets and be practical in terms of common production practices and systems (Table 1). For example, sodium bicarbonate was included in 40F and 50F as a ruminal buffering agent but not in 60F or 70F. The average CP concentration for each diet was slightly less than formulated for (i.e., 16.5%) possibly because of one or more ingredients having a level slightly lower than assumed. The predicted level of rumen undegraded intake protein ranged from 34% to 36% of total CP, and rumen degraded intake protein was projected to be between 15% and 16% of total digestible nutrient intake. Intended levels of Ca and P were 0.8% and 0.4% DM, respectively, Mg levels ranged between 0.39% and 0.43%, and the anticipated level of S was 9.2% to 10.0% of total N intake. However, average dietary levels of NDF and ADF determined from analysis of samples of 60F and 70F were appreciably greater than expected. Conversely, the predicted NDF concentrations for 40F and 50F of 39.9% and 43.1% were similar to those determined from analysis (i.e., 37.8% and 42.1%, respectively). Assuming that levels of NDF in the concentrate feedstuffs of 60F and 70F were similar to those assumed, levels of NDF in the grass hay would be near 78% and 72% to yield the analyzed dietary levels of 53.5% and 55.4%, respectively. Unfortunately, samples of grass hay in 60F and 70F were not analyzed along with those of the total mixed diets. Although care was taken in sampling of diets and formation of composite samples, one factor that could have contributed to the relatively high level of NDF in 60F and 70F is nonrepresentative sampling, which the large particle size, as noted later, could have contributed to. And, it would be difficult to attribute the considerable differences between expected and analyzed levels to this one factor. Possibly the grass sample collected for analysis by the commercial source could have been nonrepresentative as well. Nonetheless, it appears that although the grass hay source in 60F and 70F was high in fiber, this fiber, based on NDF and ADF digestibilities discussed later, was readily available to ruminal microorganisms. In support, the level of 10.13039/100016388ADL relative to NDF was much lower for the 60F and 70F than for 40F and 50F (i.e., 14.8%, 15.9%, 8.4%, and 8.7% for 40F, 50F, 60F, and 70F, respectively). Based on differences in concentrations of NDF, ADF, and ADL, cellulose was 21.3%, 23.8%, 26.7%, and 27.4% and hemicellulose was 10.9%, 11.6%, 22.3%, and 23.2% for 40F, 50F, 60F, and 70F, respectively.

For the diet particle size analysis, DM retention on the 19-mm sieve was much greater (*P <* 0.05) for 60F and 70F than for 40F and 50F, corresponding to less retention on the 8- and 4-mm sieves and lower cumulative percent undersized values for 60F and 70F (Table 2). However, particle retention on the bottom pan was similar among diets (*P* > 0.05). The concentration of peNDF based on particle retention on the three sieves was also greatest for the two diets highest in forage. However, because variation among diets based on the Bartlett test was not homogenous, standard deviations for particle size data are also presented for individual diets. Lastly, based on temperature and humidity, the temperature-humidity index increased slightly as period of the study progressed (Table S1), but not to levels to induce heat stress (Silanikove and Koluman, 2015).

**Table 2:** Particle characteristics of diets containing various levels and sources of forages fed to lactating Alpine goats based on the Penn State Particle Separator.^1^

Item^2^^,^^3^, %	*P*-value	Diet^4^				SEM
		40F	50F	60F	70F	
Mean						
Particle retention						
19-mm sieve	<0.001	10.0^a^	15.8^a^	50.1^b^	55.5^b^	3.30
8-mm sieve	<0.001	29.4^b^	28.3^b^	9.1^a^	6.3^a^	1.26
4-mm sieve	<0.001	31.8^c^	28.7^c^	16.2^b^	12.6^a^	1.11
Bottom pan	0.452	28.7	27.2	24.6	25.6	1.92
Cumulative percent undersized^3^						
8-mm sieve	<0.001	60.5^b^	55.9^b^	40.8^a^	38.2^a^	2.78
peNDF_4.0_	<0.001	26.1^a^	29.6^b^	38.3^c^	40.0^c^	1.19
Standard deviation^5^						
Particle retention						
19-mm sieve	0.001	5.96	11.18	15.66	17.05	
8-mm sieve	0.001	5.32	7.23	3.74	2.60	
4-mm sieve	0.005	3.08	2.61	5.43	5.73	
Bottom pan	0.030	5.09	5.69	9.24	9.66	
Cumulative percent undersized						
8-mm sieve	0.002	6.26	7.36	13.30	14.98	
peNDF_4.0,_	0.010	2.65	3.79	5.95	5.79	

^a–c^Means in a row without a common superscript letter differ (*P* < 0.05).

1 https://extension.psu.edu/penn-state-particle-separator; 16 weekly samples; air-dry basis.

2 Cumulative % undersized for the 19-mm sieve is the difference between 100% and particle retention on that sieve (i.e., 90.0%, 84.2%, 49.9%, and 44.5%), and that for the 4-mm sieve is the difference between 100% and particle retention on the bottom pan (i.e., 71.3%, 72.8%, 75.4%, and 74.4% for 40F, 50F, 60F, and 70F, respectively).

3 Effective or physically effective neutral detergent fiber (peNDF) is based on the sum of particle retention on the three sieves (i.e., the difference between 100% and particle retention on the bottom pan and cumulative % undersized for the 4-mm sieve) and dietary concentrations of dry matter and NDF.

4 Diets were 40%, 50%, 60%, and 70% forage (40F, 50F, 60F, and 70F, respectively), with forage in 60F and 70F diet being grass hay (primarily orchardgrass) and that in 40F and 50F cottonseed hulls, dehydrated alfalfa pellets, and wheat hay.

5 Homogeneity of variance was evaluated with the Bartlett test.

### 3.2 Digestion and gas exchange

Intake and digestibility of DM and OM immediately after the 16-wk lactation phase were similar among diets, including digestible DM and OM intake (*P >* 0.05; Table 3). Likewise, parity did not affect any of these variables. Crude protein intake was similar among diets, although digestibility of CP was greater for 60F vs. 50F (*P <* 0.05), with intermediate values for 40F and 70F (*P >* 0.05). Crude protein digestibilities were slightly greater than expected (i.e., 71.2% to 71.6%) based on 88% true protein digestibility and metabolic fecal CP of 2.67% of DM intake determined by Moore et al. (2004). Digestible CP intake was similar among diets. Intake of NDF was greater for 70F than for 40F and 50F (*P <* 0.05), with an intermediate value for 60F (*P >* 0.05). Total tract digestibility of NDF was greater for 60F and 70F than for 40F and 50F (*P <* 0.05). In this regard, NDF digestibility for 60F and 70F was much more similar to OM digestibility than values for 40F and 50F, with differences of 19.0, 15.7, 7.4, and 2.6 percentage units for 40F, 50F, 60F, and 70F, respectively. Intake of digested NDF ranked (*P <* 0.05) 40F and 50F < 60F < 70F. Intake of ADF was similar among diets (*P >* 0.05). In slight contrast to NDF digestibility, digestion of ADF ranked (*P <* 0.05) 40F and 50F < 60F < 70F, with highest intake of digested ADF among diets for 70F (*P <* 0.05). These results suggest a relatively greater difference between the two diets highest in concentrate and those highest in forage in digestion of cellulose than of hemicellulose, although again levels of hemicellulose in 60F and 70F were approximately twice as great as in 40F and 50F. The 40F and 50F diets contained cottonseed hulls, a byproduct of cotton processing, as a predominant non-forage fiber source that is high in NDF and lignin (Kononoff and Heinrichs, 2003; Zanine et al., 2023), whereas 60F and 70F contained hay primarily of orchardgrass as the only forage source. The high lignin content in cottonseed hulls was likely responsible for lower NDF and ADF digestibility for 40F and 50F than 60F and 70F. In a study comparing different fiber sources such as corn fiber, soybean hulls, oat hulls, and cottonseed hulls, total tract NDF digestibility in sheep was the lowest (80%, 72%, 41% and 27%, respectively) for the cottonseed hulls-based diet (Hsu et al., 1987). Similarly, inclusion of cottonseed hulls in the diet of sheep up to 30% reduced NDF digestibility by about 20 percentage units (Zanine et al., 2023). Respiratory gas exchange and methane production (L/d) or yield (L/kg DM or OM intake and digestible DM or OM intake) were not affected by diet, parity, or their interaction (Table 3). Forage concentration in diets usually has a positive association with methane yield (Fernández et al., 2021; Patra and Lalhriatpuii, 2016). Although the methane yield relative to digestible nutrient intake was not significant (*P* > 0.05) due to high variability in methane yield (coefficient of variation of 34%) in this study, there was a numerical difference of around 20% between 40F and 70F. Similarly, Aguerre et al. (2011) reported that methane yield as g/kg feed intake increased by 23% with increased forage levels (47% to 68%) in lactating dairy cows. Na et al. (2017), however, reported lower methane yield (g/kg DM intake) with increasing forage levels, though methane yield as g/kg digestible OM intake were similar among the goats.

**Table 3:** Effects of dietary treatment and parity on intake and digestion in lactating Alpine goats fed diets with various levels and sources of forages during the digestion trial and gas exchange during calorimetry study.

Item	Source of variation^1^			Diet^2^				SEM	Parity^3^		SEM
	TRT	PAR	TRT × PAR	40F	50F	60F	70F		1	2	
Dry matter											
Intake, g/d	0.777	0.443	0.505	1957	1829	1788	1818	125.4	1800	1896	88.7
Digestion, %	0.381	0.104	0.933	74.5	71.8	74.6	72.9	1.28	72.4	74.5	0.91
Digested, g/d	0.788	0.287	0.551	1468	1318	1337	1366	116.7	1311	1433	80.3
Organic matter											
Intake, g/d	0.750	0.442	0.498	1781	1655	1618	1654	113.8	1633	1721	80.4
Digestion, %	0.400	0.123	0.927	76.5	74.1	76.3	74.8	1.21	74.5	76.4	0.85
Digested, g/d	0.752	0.300	0.527	1370	1229	1238	1270	103.4	1223	1331	73.1
Crude protein											
Intake, g/d	0.884	0.565	0.464	313	295	311	296	20.5	298	310	14.5
Digestion, %	0.046	0.099	0.824	75.8^ab^	73.3^a^	78.2^b^	75.8^ab^	1.21	74.8	76.8	0.85
Digested, g/d	0.750	0.365	0.510	239	217	244	231	18.8	224	241	13.3
Neutral detergent fiber											
Intake, g/d	0.007	0.300	0.550	732^a^	752^a^	854^ab^	1006^b^	58.3	806	867	41.2
Digestion, %	<0.001	0.174	0.988	57.5^a^	58.4^a^	68.9^b^	72.2^b^	1.77	63.0	65.5	1.25
Digested, g/d	<0.001	0.124	0.605	426^a^	442^a^	590^b^	747^c^	51.6	516	587	36.5
Acid detergent fiber											
Intake, g/d	0.199	0.360	0.515	508	538	483	588	35.5	513	546	25.1
Digestion, %	<0.001	0.491	0.991	54.7^a^	57.1^a^	64.8^b^	71.3^c^	2.30	61.1	62.7	1.63
Digested, g/d	0.008	0.257	0.624	282^a^	307^a^	314^a^	432^b^	31.6	315	352	22.3
Gas exchange											
O_2_ consumption, L/d	0.478	0.496	0.112	586	569	548	568	17.2	562	674	12.2
CO_2_ production, L/d	0.714	0.613	0.290	572	557	543	549	19.0	551	560	13.4
CH_4_ production, L/d	0.465	0.500	0.600	38.4	39.9	44.1	40.1	2.61	39.7	41.5	1.84
CH_4_ production, L/kg DMI^4^	0.389	0.975	0.317	20.7	22.4	24.7	24.1	2.04	23.0	23.0	1.28
CH_4_ production, L/kg DDMI^4^	0.444	0.726	0.389	28.2	31.4	33.6	34.2	2.85	32.3	31.3	2.01
CH_4_ production, L/kg OMI^4^	0.377	0.973	0.311	22.7	24.7	27.3	26.5	1.98	25.3	25.3	1.40
CH_4_ production, L/kg DOMI^4^	0.417	0.756	0.377	30.2	33.6	36.2	36.5	3.00	34.5	33.6	2.12

DMI = dry matter intake; DDMI = digestible dry matter intake; OMI = organic matter intake; DOMI = digestible organic matter intake.

^a–c^Means within grouping without a common superscript letter differ (*P* < 0.05).

1 TRT = dietary treatment; PAR = parity (first vs. multiple lactations).

2 Diets were 40%, 50%, 60%, and 70% forage (40F, 50F, 60F, and 70F, respectively), with forage in 60F and 70F diet being grass hay (primarily orchardgrass) and that in 40F and 50F cottonseed hulls, dehydrated alfalfa pellets, and wheat hay.

3 Parities were first vs. multiple lactations (1 and 2, respectively).

4 Values were calculated based on the feed intake and digestibility during the fecal collection period.

### 3.3 Body weight, feed intake, and Heat energy

Body weight was similar among diets (*P =* 0.126) though numerically less for 60F and 70F than for 40F and 50F (Table 4 and Table S2). Moreover, BW was similar between parities (*P >* 0.05), and differences of small magnitude among periods were noted (*P* < 0.05). Estimates of change in BW by regressing values against time as well as based on differences between at the beginning and end of periods were quite variable and, thus, values are not presented.

**Table 4:** Effects of dietary treatment, parity, and period on dry matter intake, body weight, heart rate, heat energy, and milk composition and yield in lactating Alpine goats fed diets with various levels and sources of forages during the entire study period.

Item^1^	Dietary treatment^2^				SEM	Period^3^				SEM	Parity^4^		SEM
	40F	50F	60F	70F		1	2	3	4		1	2	
BW, kg	58.1	59.4	54.6	54.6	1.72	56.9^b^	56.0^a^	57.0^b^	56.7^b^	0.88	56.0	57.4	1.22
DMI, kg/d	2.71^b^	2.75^b^	1.96^a^	1.95^a^	0.133	2.28^ab^	2.35^b^	2.54^c^	2.20^a^	0.078	2.40	2.28	0.094
DMI, % BW	4.49	4.33	3.79	3.73	0.247	3.94^ab^	4.13^b^	4.40^c^	3.87^a^	0.141	4.15	4.02	0.171
Heart rate, beats/min	114	120	111	113	3.2	119	116	112	110	3.1	115	114	2.3
Heat energy, MJ/d	15.95	17.78	14.51	16.41	0.832	16.87	16.24	15.89	15.64	0.563	15.84	16.49	0.588
Heat energy, kJ/kg BW^0.75^	759	826	730	815	34.1	813	793	765	761	24.9	775	790	24.1
Milk composition													
Fat, %	3.16^ab^	3.37^b^	2.93^a^	2.97^a^	0.120	3.36^b^	3.00^a^	2.99^a^	3.08^a^	0.071	3.14	3.07	0.085
Protein, %	2.62^ab^	2.69^b^	2.58^ab^	2.52^a^	0.042	2.81^c^	2.57^b^	2.53^ab^	2.51^a^	0.026	2.62	2.59	0.030
Lactose, %	4.43	4.43	4.35	4.35	0.034	4.55^c^	4.39^b^	4.36^b^	4.26^a^	0.019	4.40	4.38	0.024
TS, %	10.77^ab^	11.04^b^	10.41^a^	10.41^a^	0.159	11.31^b^	10.52^a^	10.43^a^	10.38^a^	0.091	10.72	10.60	0.112
SNF, %	7.61^ab^	7.66^b^	7.48^a^	7.44^a^	0.062	7.94^c^	7.50^b^	7.44^b^	7.30^a^	0.035	7.57	7.52	0.044
Urea nitrogen, mg/dL	21.4	21.9	21.9	21.1	0.79	20.7^a^	20.9^a^	23.3^b^	21.4^a^	0.50	21.2	22.0	0.56
Log SCC, /mL	6.37	6.63	6.47	6.61	0.273	6.50^b^	6.21^a^	6.53^b^	6.85^c^	0.152	6.60	6.44	0.191
Milk yield													
Raw, kg/d	2.82^b^	2.71^b^	2.23^a^	2.10^a^	0.157	2.65^c^	2.55^b^	2.50^b^	2.18^a^	0.083	2.31^a^	2.63^b^	0.111
Fat, g/d	90.7^b^	92.3^b^	65.9^a^	62.7^a^	6.60	89.3^c^	77.4^b^	76.4^b^	68.5^a^	3.48	73.2	82.6	4.67
Protein, g/d	74.5^b^	73.5^b^	57.9^a^	52.8^a^	4.41	74.6^c^	66.0^b^	63.4^b^	54.7^a^	2.36	60.8	68.5	3.12
Energy, MJ/d	7.51^b^	7.45^b^	5.68^a^	5.34^a^	0.471	7.30^c^	6.57^b^	6.43^b^	5.67^a^	0.247	6.09	6.89	0.333
Energy, MJ/kg DMI	2.97	2.98	2.79	2.88	0.165	3.40^c^	2.88^b^	2.68^a^	2.65^a^	0.099	2.71^a^	3.09^b^	0.116
Lactose, g/d	125.9^b^	120.2^b^	97.5^a^	91.7^a^	7.13	120.8^c^	112.4^b^	109.2^b^	93.0^a^	3.79	101.4^a^	116.3^b^	5.04
TS, g/d	307.1^b^	301.0^b^	233.9^a^	219.1^a^	18.50	300.1^c^	270.2^b^	262.9^b^	227.9^a^	9.73	248.4	282.2	13.08
SNF, g/d	216.2^b^	208.4^b^	167.8^a^	156.4^a^	8.68	210.8^c^	192.0^b^	186.5^b^	159.3^a^	6.52	174.9	199.5	8.68

BW = body weight; DMI = dry matter intake; TS = total solids; SNF = solids-non-fat; SCC = somatic cell count.

^a–f^Means within grouping without a common superscript letter differ (*P* < 0.05).

1 *P*-values and interaction effects associated with these variables have been presented in Tables S2 and S3.

2 Diets were 40%, 50%, 60%, and 70% forage (40F, 50F, 60F, and 70F, respectively), with forage in 60F and 70F diet being grass hay (primarily orchardgrass) and that in 40F and 50F cottonseed hulls, dehydrated alfalfa pellets, and wheat hay.

3 Periods were 28 d in length.

4 Parities were first vs. multiple lactations (1 and 2, respectively).

During the entire lactation phase, DM intake was less for 60F and 70F vs. 40F and 50F (*P <* 0.05). Intake of DM in both kg/d and % BW peaked in period 3 and then declined slightly in period 4. However, there was an interaction between diet and period (*P <* 0.05), with differences for 40F and 50F vs. 60F and 70F slightly greater in periods 1 and 2 than in periods 3 and 4 (Table S3). Intake of DM in percent of BW also differed among diets in a manner similar to kg/d, although the dietary treatment main effect only approached significance (*P =* 0.071). Relatively low feed intake of the two high-forage diets might be attributable to differences in physical structure of the forage sources, namely the relatively large particle size of forage and higher peNDF contents in 60F and 70F rather than differences in rate of digestion and, thus, passage rate (Shaver et al., 1986; Tafaj et al., 2007) since OM digestibility was similar among the diets. This is supported by greater time spent in eating per unit of NDF intake as described later.

Heart rate was not affected by diet, period, or parity (*P >* 0.05; Table 4 and Table S2). Likewise, main effects of diet, period, and parity for HE in MJ/d and kJ/kg BW^0.75^ were not significant (*P >* 0.05), but there were diet × period interactions (*P* < 0.05). This was largely because of high values for 50F in periods 1 and 2 relative to periods 3 and 4 (Table S3).

### 3.4 Milk yield and components

Milk fat concentration was greater for 50F than for 60F and 70F (*P <* 0.05), with an intermediate value for 40F (*P* < 0.05; Table 4 and Table S2). The milk fat level was highest among periods for period 1 (*P <* 0.05) and similar between parities (*P >* 0.05). Results were fairly similar for milk protein concentration. The milk lactose concentration was not affected by main effects of diet or parity (*P >* 0.05), but there were diet × period and parity × period interactions (*P <* 0.05; Table S2). Concentrations of total solids and solids-not-fat were greater for 50F than for 60F and 70F (*P <* 0.05), with intermediate values for 40F (*P >* 0.05). The concentration of total solids was greatest among periods for period 1 (*P <* 0.05), and the ranking for solids-not-fat was period 1 > 2 and 3 > 4. The urea N concentration was similar among diets and between parities (*P >* 0.05) and greatest among periods for period 3 (*P <* 0.05). The log of SCC also was similar among diets and between parities (*P >* 0.05) and ranked (*P* < 0.05) period 2 < 1 and 3 < 4. One of the postulates regarding use of 60F and 70F was that the high dietary levels of ruminally degradable fiber and peNDF content in Alpine goats would result in greater fiber degrading microbial populations and ruminal concentration of acetate (Patra and Yu, 2015; Xue et al., 2022), which would be expected to lead to increases in milk fat concentration and yield (Seymour et al., 2005; Urrutia et al., 2019). The molar percentage of acetate in ruminal fluid was in fact increased, but milk fat concentration and yield were not, rather being lower for the two diets highest in fiber. Although exact reasons why these expectations were not realized are uncertain, relatively low feed intake, consequently probably lower metabolizable energy intake (because digestibility and heat production were similar among treatments) for 60F and 70F probably was a major contributing factor. As noted elsewhere, this appears to have involved the large particle size and greater peNDF content of 60F and 70F rather than limited digestibility. Similar to fat concentration and yield, protein and energy concentrations and their yields were lower for 60F and 70F, which were presumably due to lower amount of feed intake (Schmidely et al., 1999; Silva et al., 2018).

Raw milk yield was greater for 40F and 50F than for 60F and 70F, ranked period 1 > 2 and 3 > 4, and was greater for does vs. doelings (*P <* 0.05; Table 4). However, there were interactions between diet and period and between parity and period (*P <* 0.05; Table S2). The diet × period interaction was due in part to little change in periods 1 through 3 for 40F relative to other diets, particularly 60F and 70F (Table S3). The parity × period interaction was mainly because of less change with advancing time for doelings relative to does. Similar differences and interactions were noted for yields of the major milk constituents. However, it is interesting to note that milk energy yield relative to DM intake did not differ among diets (*P >* 0.05). Also, this ratio averaged 15.5% greater for does than for doelings (*P* < 0.05).

The similar milk energy yield to feed intake ratio among diets, along with no differences in OM digestibility, suggest that the efficiency of dietary energy utilization for milk production was similar among diets, with differences in yield of milk and its components related to level of feed intake. Again, the 60F and 70F diets were high in fiber, but that was of relatively high digestibility. Thus, it would appear that an important constraining factor to feed intake and milk yield for the two high-forage diets involved the physical structure, namely the relatively large particle size. The primary purpose or function of ingestive mastication is to increase the surface area of ingesta to facilitate rapid attachment of ruminal microorganisms to ingested particles and greater accessibility to microbial enzymes (Luginbuhl et al., 1989; Pond et al., 1984) to minimize lag time and increase potential DM and NDF digestion (Beauchemin, 1992), which appears to have been achieved based on the digestibility for the high forage diets. Ruminative mastication occurs largely to reduce particle size and facilitate change in specific gravity so that digesta exits through the reticulo-omasal orifice to lessen ruminal digesta fill, reduce stimulation of stretch receptors in the rumen wall, and thereby allow for ingestion of additional feed (Beauchemin, 2018; Deswysen and Ellis, 1990). Based on these findings and postulates, it would be of interest to conduct a similar study with more grinding of 60F and 70F to decrease initial size of prehended particles that conceivably would increase level of feed intake.

Factors responsible for the greater ratio of milk energy yield to DM intake for does vs. doelings are not entirely clear. However, OM digestibility and digestible OM intake were numerically greater for does, as was also the case for milk energy yield in MJ/d (*P =* 0.093), and BW was similar between parities. Hence, a factor that could have contributed to the difference in this ratio is energy and nutrients required for development and function of the mammary gland of doelings given that this was the first lactation. And, although this is not predicted in reports of Nsahlai et al. (2004) or Sahlu et al. (2004), as well as in energy and nutrient requirement recommendations such as of NRC (2007) for goats, in some systems maintenance energy requirements decrease in a continuous fashion with advancing age (e.g., sheep of NRC, 2007).

### 3.5 Ruminal fluid characteristics

There were numerous interactions in ruminal fluid variables (*P* < 0.05) that makes a simple and clear presentation and interpretation of data challenging (Table S4). Therefore, main effect means for diet, period, and parity are first presented in Table 5 and addressed before attention is given to interactions.

**Table 5:** Effects of dietary treatment, period, and parity on ruminal fluid characteristics of Alpine doelings in the first lactation and does fed diets with various levels and sources of forages.

Item^1^	Diet^2^				SEM	Period^3^				SEM	Parity^4^		SEM
	40F	50F	60F	70F		1	2	3	4		1	2	
pH	5.75^a^	5.72^a^	5.91^b^	5.97^b^	0.035	5.97^c^	5.85^b^	5.77^a^	5.77^a^	0.022	5.84	5.84	0.025
Total volatile fatty acids, mM	64.8	67.3	63.4	62.7	1.49	71.7^d^	68.6^c^	64.8^b^	53.1^a^	1.21	64.7	64.4	1.06
Acetate, %	66.3^a^	67.2^a^	69.9^b^	71.9^c^	0.65	67.1^a^	68.0^b^	68.8^c^	71.3^d^	0.40	69.3	68.3	0.46
Propionate, %	15.8	15.4	16.6	16.2	0.34	16.5^c^	16.9^c^	15.8^b^	14.8^a^	0.23	15.9	16.0	0.24
Isobutyrate, %	0.77^b^	0.73^ab^	0.72^a^	0.69^a^	0.017	0.82^c^	0.72^b^	0.73^b^	0.64^a^	0.018	0.72	0.74	0.012
Butyrate, %	14.8^b^	14.4^b^	10.8^a^	9.3^a^	0.68	13.2^c^	12.3^b^	12.5^b^	11.3^a^	0.38	11.9	12.8	0.48
Isovalerate, %	1.16^b^	1.08^ab^	1.05^a^	0.98^a^	0.035	1.18^c^	1.03^ab^	1.08^b^	0.97^a^	0.033	1.06	1.07	0.025
Valerate, %	1.18^b^	1.16^b^	0.96^a^	0.90^a^	0.040	1.14^c^	1.07^b^	1.07^b^	0.92^a^	0.024	1.01	1.09	0.029
Acetate:propionate	4.38	4.51	4.31	4.52	0.106	4.15^a^	4.16^a^	4.44^b^	4.97^c^	0.072	4.45	4.41	0.075
Ammonia nitrogen, mg/dL	23.3	23.1	23.0	22.8	0.93	23.0^ab^	23.5^b^	21.9^a^	23.8^b^	0.61	23.0	23.1	0.66

^a–d^Means within grouping without a common superscript letter differ (*P* < 0.05).

1 *P*-values and interaction effects associated with these variables have been presented in Tables S4, S5, S6, S7, and S8.

2 Diets were 40%, 50%, 60%, and 70% forage (40F, 50F, 60F, and 70F, respectively), with forage in 60F and 70F diet being grass hay (primarily orchardgrass) and that in 40F and 50F cottonseed hulls, dehydrated alfalfa pellets, and wheat hay.

3 Periods were 28 d in length.

4 Parities were first vs. multiple lactations (1 and 2, respectively).

Ruminal pH was slightly lower for 40F and 50F than for 60F and 70F (*P <* 0.05; Table 5), as might be expected based on differences in dietary ingredient levels. Period affected ruminal pH as it did for all other ruminal fluid measures (*P <* 0.05). Factors responsible for these period differences are not readily apparent. In contrast, no ruminal fermentation measure was influenced by parity (*P >* 0.05). The molar percentage of acetate ranked (*P <* 0.05) 40F and 50F < 60F < 70F, although there were no differences among diets in the molar percentage of propionate or the ratio of acetate to propionate. A higher proportion of acetate and acetate to propionate ratio for 60F and 70F were expected (Patra and Yu, 2015). High fiber diets promote rumen microbial production of acetate and its conversion to butyrate through the two acetyl-CoA to acetoacetyl-CoA pathway by microorganisms such as *Lachnospira* spp. and *Butyrivibrio* spp. (Li et al., 2022), but it does not seem that conversion of acetate to butyrate was elevated with the 60F and 70F diets, which was in agreement with other studies addressing different dietary forage levels (Murphy et al., 2000; Penner et al., 2009; Zhang et al., 2017). In fact, butyrate production in the rumen depends upon the balance of dietary concentrations of degradable fiber and starch, which influences direct use by some lactate-producing bacteria of acetate for butyrate production via butyryl-CoA/acetate-CoA transferase rather than through the acetoacetyl-CoA pathway (Diez-Gonzalez et al., 1999; Plöger et al., 2012). In the current study, perhaps the level of nonfiber carbohydrate in the high-fiber was not optimal to stimulate the direct pathway of butyrate production. Levels of minor VFA of isobutyrate, isovalerate, and valerate were either lower for 60F and 70F than for 40F and 50F or tended to differ, with their production from deamination of amino acids by ruminal microorganisms (Apajalahti et al., 2019). The high-fiber diets included modified expeller soybean meal (Soyplus; Dairy Nutrition Plus, Ralston, IA, USA), whereas the low-fiber diets contained soybean meal as a major protein source, with considerable differences in ruminally degradable protein content (e.g., 600 vs. 350 g/kg; Vendramini et al., 2011). The greater content of ruminal undegradable protein in 60F and 70F vs. 40F and 50F may account for the differences in concentrations of these minor VFA. The greatest diet effects were observed for the molar percentage of butyrate, with values much less for 60F and 70F than for 40F and 50F (*P* < 0.05). The ruminal ammonia N concentration was similar among diets (*P* > 0.05).

Interactions in ruminal fluid measures are addressed in Tables S5–S8. First, differences in pH among diets varied with period (Table S5). Ruminal pH was less for the PM vs. AM sampling time (*P* < 0.05), with the magnitude of difference varying among periods (Table S5). Although this sampling time difference would seem related to time taken for microbial adhesion to particles and the onset of enzymatic breakdown, there was not a diet × time interaction as might have been expected based on initial particle size of diets ingested. Differences in the acetate to propionate ratio between times of sampling were observed with 40F and 50F but not 60F or 70F.

For doelings, the magnitude of differences among diets in total VFA concentration was greater in period 1 than in other periods (Tables S6 and S7). The molar percentage of acetate was generally greater for 60F and 70F than for 40F and 50F, but again differences varied among periods. The molar percentage of propionate was not influenced by any interaction (*P >* 0.05), ranked (*P <* 0.05) periods 1 and 2 > 3 > 4, and was greater for AM vs. PM (*P <* 0.05). The greatest value among diet × period means of the molar percentage of butyrate was for 50F-period 1 (*P* < 0.05). Diet main effect means of the ruminal ammonia N concentration as well as diet × period means indicate that ruminally available N was not limiting to microbial growth and digestion throughout the lactation phase (Hoover, 1986). Differences among diet means for ruminal ammonia N concentration were not consistent among periods.

In contrast to doelings, the concentration of total VFA in ruminal fluid of does was not affected by diet (*P >* 0.05; Tables S6 and S8). The total VFA concentration was lowest among period × time means for period 4-AM (*P <* 0.05). The molar percentage of acetate was greater for 70F than for 40F and 50F and for 60F vs. 40F (*P <* 0.05). The level was greatest among periods for period 4 (*P* < 0.05). The molar percentage of propionate was generally less in period 4 than periods 1 and for 60F and 70F. As was the case for doelings, the molar percentage of butyrate was in most cases less for the two diets highest in forage than for the diets higher in concentrate. As for doelings, concentrations of ruminal ammonia N for does were greater than commonly assumed required for unhindered microbial activity.

### 3.6 Blood constituent concentrations

In contrast to ruminal fluid measures, there was only one blood constituent variable for which a significant interaction existed (i.e., cholesterol; Table 6, Tables S9 and S10). The total protein concentration was similar among diets and between parities (*P >* 0.05), with significant but relatively small differences among periods (*P <* 0.05). In accordance, albumin concentration was not affected by diet, period, or parity (*P >* 0.05). The degree to which the cholesterol concentration was lower for 70F than for 40F and 50F varied among periods, with greater values for 60F than for 70F in periods 1 and 3 but not 2 and 4 (Table S10). The lower concentration of cholesterol for the high-fiber diets might be attributed to lower energy intake and reduced lipogenesis (Liu et al., 2020). Diet and parity did not affect levels of triglycerides or glucose (*P >* 0.05). The triglyceride concentration was lowest among periods for period 1 (*P <* 0.05), and the level of glucose was greater in period 3 than in periods 1 and 4 (*P <* 0.05). The blood lactate concentration was lower for 60F than for 40F and 50F (*P <* 0.05), with an intermediate value for 70F (*P >* 0.05). In general, the higher blood lactate level in for the low-fiber diets was perhaps due to greater production and absorption of lactate in the rumen (Brown et al., 2006; Serment et al., 2011). The level was less in periods 1 and 2 vs. 3 and 4 and for does vs. doelings (*P* < 0.05).

**Table 6:** Effects of dietary treatment, period, and sampling time on blood constituent concentrations in lactating Alpine goats fed diets with various levels and sources of forages.

Item^1^	Diet^2^				SEM	Period^3^				SEM	Parity^4^		SEM
	40F	50F	60F	70F		1	2	3	4		1	2	
Total protein, g/dL	8.07	7.91	7.86	7.80	0.133	7.85^ab^	7.97^bc^	7.71^a^	8.09^c^	0.084	7.85	7.97	0.098
Albumin, g/dL	2.96	2.84	2.86	2.90	0.047	2.87	2.88	2.89	2.91	0.029	2.89	2.89	0.033
Cholesterol, mg/dL	139^c^	134^bc^	121^ab^	105^a^	6.0	115^a^	127^b^	126^b^	130^b^	3.4	119	131	4.3
Triglycerides, mg/dL	33.4	33.1	31.8	32.1	1.19	29.8^a^	33.3^b^	34.4^b^	32.9^b^	0.93	32.7	32.5	0.84
Glucose, mg/dL	56.1	57.8	54.9	55.0	0.98	53.4^a^	56.9^bc^	58.0^c^	55.5^b^	0.75	56.6	55.3	0.69
Lactate, mg/dL	4.79^b^	4.80^b^	4.00^a^	4.14^ab^	0.240	4.01^a^	4.18^a^	4.86^b^	4.68^b^	0.172	4.92^b^	3.94^a^	0.170

^a–c^Means within grouping without a common superscript letter differ (*P* < 0.05).

1 *P*-values and interaction effects associated with these variables have been presented in Tables S9 and S10.

2 Diets were 40%, 50%, 60%, and 70% forage (40F, 50F, 60F, and 70F, respectively), with forage in 60F and 70F diet being grass hay (primarily orchardgrass) and that in 40F and 50F cottonseed hulls, dehydrated alfalfa pellets, and wheat hay.

3 Periods were 28 d in length and samples were collected at 3 to 4 h after feeding in the morning.

4 Parities were first vs. multiple lactations (1 and 2, respectively).

### 3.7 Body condition score, linear measures, and BMI

Body condition score was lower for 60F and 70F than for 40F and 50F (*P <* 0.05), greatest among periods for period 4 (*P <* 0.05), and similar between parities (*P >* 0.05; Table 7 and Table S11). Body condition score is reflective of body fat stores, especially in the subcutaneous areas (Liu et al., 2019; Wang et al., 2022); therefore, lower BCS for the high-fiber diets may relate to lower feed intake (Belkasmi et al., 2023; Lourencon et al., 2023). The only linear measure affected by diet was heart girth, being greater for 40F and 50F than for 60F and 70F (*P <* 0.05). Presumably this relates to greater feed intake along with numerically greater BW for the two diets highest in concentrate. The four BMI evaluated were lower or tended to be less for 60F and 70F than for 40F and 50F. There were period differences for each of the BMI (*P <* 0.05) but none between parities (*P* > 0.05). The BMI is perhaps more reflective of whole body condition and impacted by less subjectivity compared with BCS, which is more reflective of subcutaneous fat (Liu et al., 2019; Wang et al., 2022). However, from the current and other studies (Belkasmi et al., 2023; Lourencon et al., 2023), it appears that BCS is a more sensitive tool than BMI to assess nutritional status in sheep and goats. Body condition score, BMI, and BW suggest that relatively more energy and nutrients were available for use by tissues in addition to the mammary gland with 40F and 50F vs. 60F and 70F.

**Table 7:** Effects of dietary treatment, parity, and period on body condition score, linear measures, and body mass indexes in lactating Alpine goats fed diets with various levels and sources of forages.

Item^1^^,^^2^	Dietary treatment^3^				SEM	Period^4^				SEM	Parity^5^		SEM
	40F	50F	60F	70F		1	2	3	4		1	2	
BCS	2.83^b^	2.89^b^	2.65^a^	2.53^a^	0.060	2.72^b^	2.64^a^	2.69^ab^	2.85^c^	0.035	2.69	2.75	0.042
Hook, cm	59.7	59.2	50.6	58.5	0.50	59.4	59.5		58.9	0.32	59.3	59.2	0.35
Pin, cm	75.2	75.0	74.1	73.6	0.61	70.6^a^	76.1^b^		76.8^b^	0.41	74.5	74.5	0.43
Heart girth, cm	95.1^b^	95.2^b^	92.8^a^	92.5^a^	0.81	94.0^ab^	94.3^b^		93.4^a^	0.57	94.1	93.8	0.57
Wither, cm	74.4	74.8	74.5	74.9	0.49	73.7^a^	75.6^c^		74.6^b^	0.35	74.2	75.1	0.35
Rump, cm	20.5	20.4	20.0	19.8	0.21	19.9^a^	20.0^a^		20.6^b^	0.13	20.2	20.2	0.15
BMI-WH, g/cm^2^	12.93^bc^	13.17^c^	11.85^a^	12.18^ab^	0.281	13.02^c^	12.50^b^		12.07^a^	0.157	12.46	12.60	0.198
BMI-WP, g/cm^2^	10.28^bc^	10.41^c^	9.55^a^	9.73^ab^	0.215	10.97^c^	9.77^b^		9.25^a^	0.121	9.95	10.03	0.152
BMI-GH, g/cm^2^	10.11^bc^	10.33^c^	9.51^a^	9.85^ab^	0.169	10.19^b^	10.03^b^		9.64^a^	0.100	9.82	10.08	0.120
BMI-GP, g/cm^2^	8.04^b^	8.17^b^	7.66^a^	7.86^ab^	0.128	8.58^c^	7.83^b^		7.39^a^	0.076	7.84	8.03	0.091

^a–c^Means within grouping without a common superscript letter differ (*P* < 0.05).

1 BCS = body condition score (1–5); BMI = body mass index; Wither = height at withers; Hook = point of the shoulder to hook bone; Pin = point of the shoulder to pin bone; Rump = width at hook bones; BMI-WH = BW/(Wither × Hook); BMI-WP = BW/(Wither × Pin); BMI-GH = BW/(Heart girth × Hook); BMI-GP = BW/(Heart girth × Pin).

2 *P*-values associated with these variables have been presented in Table S11.

3 Diets were 40%, 50%, 60%, and 70% forage (40F, 50F, 60F, and 70F, respectively), with forage in 60F and 70F diet being grass hay (primarily orchardgrass) and that in 40F and 50F cottonseed hulls, dehydrated alfalfa pellets, and wheat hay.

4 Periods were 28 d in length. Body condition score was assessed near the end of each period. Linear measures occurred at the beginning, middle, and end of the study, with associated body mass indexes based on BW determined at these times (periods 1, 2, and 4, respectively). Linear measures were not determined in Period 3.

5 Parities were first vs. multiple lactations (1 and 2, respectively).

### 3.8 Feeding behavior

**Fig. 1:**
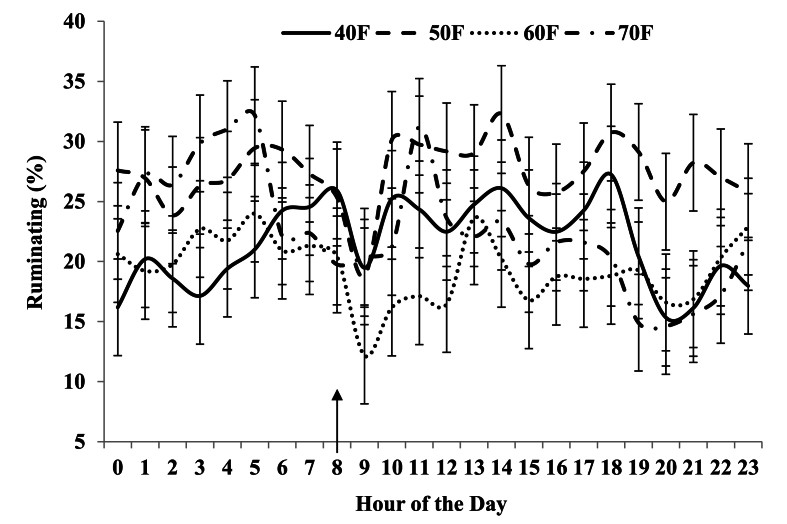
Effects of dietary treatment and hour on ruminating time of Alpine doelings in the first lactation fed diets with various levels and sources of forages. An arrow showing at the x-axis indicates the feed delivery in the Calan gate feeders. Diets were 40%, 50%, 60%, and 70% forage (40F, 50F, 60F, and 70F, respectively), with forage in 60F and 70F diet being grass hay (primarily orchardgrass) and that in 40F and 50F cottonseed hulls, dehydrated alfalfa pellets, and wheat hay.

**Fig. 2:**
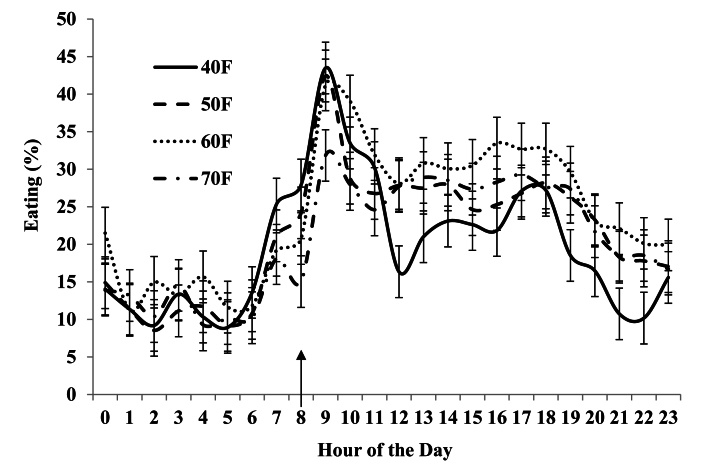
Effects of dietary treatment and hour on eating time of Alpine does fed diets with various levels and sources of forages. An arrow showing at the x-axis indicates the feed delivery in the Calan gate feeders. Diets were 40%, 50%, 60%, and 70% forage (40F, 50F, 60F, and 70F, respectively), with forage in 60F and 70F diet being grass hay (primarily orchardgrass) and that in 40F and 50F cottonseed hulls, dehydrated alfalfa pellets, and wheat hay.

Main effects of diet, period, and parity did not influence time spent ruminating or idle (*P >* 0.05; Table 8 and Tables S12–S15). The same was true for eating time, except for a greater value in period 4 vs. 2 (*P <* 0.05). However, there was a diet × parity interaction in rumination and a three-way interaction in eating (*P <* 0.05; Tables S12–S15 and Figs. S1–S7). Treatment × hour interactions were significant (*P <* 0.05) for rumination in doelings (Tables S14 and S15; Fig. 1) and eating in does (Tables S14 and S15; Fig. 2). The dietary fiber content and its physical and chemical characteristics affect ruminating and eating behaviors in ruminants (Llonch et al., 2020; Mirzaei-Alamouti et al., 2021). The lack of consistent effect of diet on ingestive behaviors was not expected. That is, with the considerably greater intake of total NDF and peNDF for 60F and 70F than for 40F and 50F, it was anticipated that time spent eating and(or) ruminating would be greater and time idle would be less for the two diets highest in fiber (Adin et al., 2009; Gentry et al., 2016; Llonch et al., 2020; Zhao et al., 2011). Perhaps this is in accordance with relatively high fiber fermentability for 60F and 70F and could even reflect high fragility of ingested forage particles and considerable susceptibility to particle breakdown upon mastication compared with cottonseed hulls and wheat hay in 40F and 50F in accordance with lower rumination time per unit of NDF intake for 60F and 70F. Future studies of this nature could include particle size analysis of digesta to evaluate such postulates as conducted with duodenal digesta and feces in studies with cattle consuming diets differing in physical characteristics (Stokes et al., 1988a,b) and fermentability of peNDF in the rumen (Zebeli et al., 2006). Diet did not affect position variables, time spent standing and lying, expressed on a daily basis (*P* > 0.05; Table 8 and Table S13). But there were many differences among periods, hour, and interaction effects for time spent standing, time spent lying on the left and right sides (Table S14) and have been presented in supplementary figures (Figs. S8, S9, and S10), for which the reasons are unclear.

**Table 8:** Effects of dietary treatment, parity, and period on ingestive behavior and position on a daily basis in lactating Alpine goats fed diets with various levels and sources of forages.

Item^1^	Dietary treatment^2^				SEM	Period^3^				SEM	Parity^4^		SEM
	40F	50F	60F	70F		1	2	3	4		1	2	
Ingestive behavior, % day													
Rumination	24.0	21.7	21.9	23.2	2.21		23.7		21.8	1.56	23.1	22.3	1.56
Eating	22.7	21.1	22.6	23.0	1.58		19.8^a^		24.9^b^	1.01	23.2	21.5	1.12
Idle	53.3	57.2	55.5	53.9	2.66		56.6		53.4	1.88	53.7	56.3	1.88
Position, % day													
Standing	29.6	29.4	28.3	32.6	2.79	38.6^b^	24.6^a^	28.0^a^	28.8^a^	2.26	31.8	28.2	1.97
Lying	70.4	70.6	71.7	67.3	2.79	61.4^a^	75.4^b^	72.0^b^	71.2^b^	2.26	68.2	71.8	1.97

^a–d^Means within grouping without a common superscript letter differ (*P* < 0.05).

1 *P*-values and interaction effects have been presented in Tables S12, S13, S14 and S15.

2 Diets were 40%, 50%, 60%, and 70% forage (40F, 50F, 60F, and 70F, respectively), with forage in 60F and 70F diet being grass hay (primarily orchardgrass) and that in 40F and 50F cottonseed hulls, dehydrated alfalfa pellets, and wheat hay.

3 Periods were 28 d in length. Ingestion behavior was not measured in Period 1 and 3.

4 Parities were first vs. multiple lactations (1 and 2, respectively).

## 4 Conclusions

Diets containing relatively high levels of forage (i.e., 60% and 70%) with apparently high fiber digestibility resulted in lower milk production and energy yield in early and mid-lactation Alpine dairy goats compared to diets containing low levels of forage with low fiber digestibility (i.e., 40% and 50% forage) regardless of parity. This result appeared due primarily to limited intake of the two high-forage diets, possibly because of relatively large particle size. The high-fiber diets did not result in greater concentrations of milk fat despite of a greater molar proportion of acetate proportion in ruminal fluid. Ingestive behavior of ruminants is probably not only affected by particle size and physically effective fiber concentration of forages but also depends upon fermentability of diets. Future research should consider other high-quality forage sources, smaller diet particle size, later stages of lactation, and breeds yielding milk higher in fat and protein concentrations.

## Author contributions

**Arthur L. Goetsch:** conceptualization, methodology, validation, formal analysis, investigation, resources, data curation, writing—original draft preparation, writing—review and editing, supervision, project administration, funding acquisition. **Ryszard Puchala:** conceptualization, methodology, validation, formal analysis, investigation, resources, data curation, writing—original draft preparation, writing—review and editing, supervision, funding acquisition. **Raquel V. Lourencon:** conceptualization, methodology, formal analysis, investigation, data curation, writing—original draft preparation. **Luana P. S. Ribeiro:** methodology, investigation. **Amlan K. Patra:** validation, formal analysis, data curation, writing—original draft preparation, writing—review and editing. **Terry A. Gipson:** validation, formal analysis, investigation. **Wei Wang:** investigation. All authors have read and agreed to the published version of the manuscript.

## Declaration of competing interest

We declare that we have no financial and personal relationships with other people or organizations that can inappropriately influence our work, and there is no professional or other personal interest of any nature or kind in any product, service and/or company that could be construed as influencing the content of this paper.
