# Supplementary material for: Effects of the level and source of dietary physically effective fiber on feed intake, nutrient utilization, heat energy, ruminal fermentation, and milk production by Alpine goats
Source: Anim Nutr. 2024 Mar 4;17:312–24. doi: 10.1016/j.aninu.2024.02.002 (PMC11127095; doi:10.1016/j.aninu.2024.02.002)
Supplement: Multimedia component 1 [file mmc1.docx]

| \| **Table S1**  Average daily temperature (T), relative humidity (RH), and temperature-humidity index (THI) in the facility during four different periods of the study^1,2^. \| \| \| \| \| \| \| --- \| --- \| --- \| --- \| --- \| --- \| \| Period \| Item \| Mean \| SEM \| Minimum \| Maximum \| \| 1 \| T, ºC \| 20.9 \| 0.41 \| 15.6 \| 24.7 \| \|  \| RH, % \| 64.4 \| 2.03 \| 41.6 \| 81.8 \| \|  \| THI \| 67.3 \| 0.60 \| 59.5 \| 71.3 \| \| 2 \| T, ºC \| 23.8 \| 0.47 \| 18.3 \| 27.0 \| \|  \| RH, % \| 70.3 \| 1.19 \| 59.0 \| 79.4 \| \|  \| THI \| 72.1 \| 0.72 \| 63.5 \| 77.1 \| \| 3 \| T, ºC \| 27.6 \| 0.36 \| 24.7 \| 31.2 \| \|  \| RH, % \| 70.1 \| 1.12 \| 56.8 \| 80.1 \| \|  \| THI \| 77.7 \| 0.57 \| 72.3 \| 83.0 \| \| 4 \| T, ºC \| 30.3 \| 0.26 \| 28.1 \| 32.7 \| \|  \| RH, % \| 64.0 \| 1.21 \| 53.1 \| 74.8 \| \|  \| THI \| 80.8 \| 0.43 \| 76.4 \| 85.2 \| \| ^1^THI = (0.8 × T) + [(RH/100) × (T – 14.4)] + 46.4 (Amundson et al., 2006).  ^2^Based on daily means and two measurement locations. \| \| \| \| \| \|   **Table S2**  *P*-values for effects of dietary treatment, parity, and period on dry matter intake, body weight, heart rate, heat energy, and milk composition and yield in lactating Alpine goats fed diets with various levels and sources of forages. | | | | | | | |
| --- | --- | --- | --- | --- | --- | --- | --- | --- | --- | --- | --- | --- | --- | --- | --- | --- | --- | --- | --- | --- | --- | --- | --- | --- | --- | --- | --- | --- | --- | --- | --- | --- | --- | --- | --- | --- | --- | --- | --- | --- | --- | --- | --- | --- | --- | --- | --- | --- | --- | --- | --- | --- | --- | --- | --- | --- | --- | --- | --- | --- | --- | --- | --- | --- | --- | --- | --- | --- | --- | --- | --- | --- | --- | --- | --- | --- | --- | --- | --- | --- | --- | --- | --- | --- | --- | --- | --- | --- | --- | --- | --- | --- | --- | --- | --- | --- | --- |
| Item | Source of variation^1^ | | | | | | |
|  | TRT | PAR | TRT × PAR | PRD | TRT × PRD | PAR × PRD | TRT × PAR × PRD |
| BW, kg | 0.126 | 0.420 | 0.298 | 0.011 | 0.972 | 0.491 | 0.384 |
| DMI, kg/d | <0.001 | 0.382 | 0.094 | <0.001 | 0.009 | 0.600 | 0.439 |
| DMI, % BW | 0.071 | 0.603 | 0.021 | <0.001 | 0.140 | 0.694 | 0.607 |
| Heart rate, beats/min | 0.234 | 0.657 | 0.280 | 0.231 | 0.106 | 0.818 | 0.902 |
| Heat energy, MJ/d | 0.061 | 0.438 | 0.569 | 0.244 | 0.018 | 0.906 | 0.866 |
| Heat energy, kJ/kg BW^0.75^ | 0.158 | 0.650 | 0.766 | 0.268 | 0.036 | 0.859 | 0.887 |
| Milk composition |  |  |  |  |  |  |  |
| Fat, % | 0.049 | 0.584 | 0.503 | <0.001 | 0.108 | 0.673 | 0.999 |
| Protein, % | 0.047 | 0.468 | 0.830 | <0.001 | 0.680 | 0.276 | 0.979 |
| Lactose, % | 0.181 | 0.704 | 0.373 | <0.001 | 0.014 | 0.026 | 0.153 |
| TS, % | 0.017 | 0.477 | 0.367 | <0.001 | 0.141 | 0.884 | 0.977 |
| SNF, % | 0.044 | 0.472 | 0.357 | <0.001 | 0.121 | 0.055 | 0.727 |
| Urea nitrogen, mg/dL | 0.852 | 0.340 | 0.676 | <0.001 | 0.154 | 0.404 | 0.584 |
| Log SCC, /mL | 0.887 | 0.548 | 0.870 | <0.001 | 0.238 | 0.262 | 0.247 |
| Milk yield |  |  |  |  |  |  |  |
| Raw, kg/d | 0.004 | 0.042 | 0.399 | <0.001 | 0.002 | <0.001 | 0.946 |
| Fat, g/d | 0.002 | 0.161 | 0.395 | <0.001 | 0.007 | 0.001 | 0.996 |
| Protein, g/d | 0.001 | 0.086 | 0.421 | <0.001 | 0.026 | <0.001 | 0.882 |
| Energy, MJ/d | 0.002 | 0.093 | 0.386 | <0.001 | 0.003 | <0.001 | 0.965 |
| Energy, MJ/kg DM intake | 0.830 | 0.024 | 0.225 | <0.001 | <0.001 | 0.150 | 0.575 |
| Lactose, g/d | 0.002 | 0.042 | 0.412 | <0.001 | 0.002 | <0.001 | 0.907 |
| TS, g/d | 0.001 | 0.073 | 0.389 | <0.001 | 0.002 | <0.001 | 0.923 |
| SNF, g/d | 0.002 | 0.050 | 0.411 | <0.001 | 0.004 | <0.001 | 0.900 |
| BW = body weight; DM = dry matter; DMI = DM intake; TS = total solids; SNF = solids-non-fat; SCC = somatic cell count.  ^1^TRT = dietary treatment; PAR = parity (first vs. multiple lactations); PRD = period. | | | | | | | |

| **Table S3**  Effects of dietary treatment, parity, and period on dry matter intake, body weight, heart rate, heat energy, and milk composition and yield. | | | | | | | | | | | | | | | |
| --- | --- | --- | --- | --- | --- | --- | --- | --- | --- | --- | --- | --- | --- | --- | --- |
| Item | PRD | Dietary treatment^1^ | | | | SEM | PAR | Period^2^ | | | | SEM | Parity^3^ | | SEM |
|  |  | 40F | 50F | 60F | 70F |  |  | 1 | 2 | 3 | 4 |  | 1 | 2 |  |
| BW, kg |  | 58.1 | 59.4 | 54.6 | 54.6 | 1.72 |  | 56.9^b^ | 56.0^a^ | 57.0^b^ | 56.7^b^ | 0.88 | 56.0 | 57.4 | 1.22 |
| DMI, kg/d |  | 2.71^b^ | 2.75^b^ | 1.96^a^ | 1.95^a^ | 0.133 |  | 2.28^ab^ | 2.35^b^ | 2.54^c^ | 2.20^a^ | 0.078 | 2.40 | 2.28 | 0.094 |
|  | 1 | 2.73^c^ | 2.86^c^ | 1.76^a^ | 1.77^ab^ | 0.156 |  |  |  |  |  |  |  |  |  |
|  | 2 | 2.76^c^ | 2.78^c^ | 1.97^ab^ | 1.88^ab^ |  |  |  |  |  |  |  |  |  |  |
|  | 3 | 2.91^c^ | 2.96^c^ | 2.15^b^ | 2.14^ab^ |  |  |  |  |  |  |  |  |  |  |
|  | 4 | 2.42^b^ | 2.39^b^ | 1.98^ab^ | 1.99^ab^ |  |  |  |  |  |  |  |  |  |  |
| DMI, % BW |  | 4.49 | 4.33 | 3.79 | 3.73 | 0.247 |  | 3.94^ab^ | 4.13^b^ | 4.40^c^ | 3.87^a^ | 0.141 | 4.15 | 4.02 | 0.171 |
|  |  | 4.68^bc^ | 4.97^c^ | 3.64^a^ | 3.31^a^ | 0.340 | 1 |  |  |  |  |  |  |  |  |
|  |  | 4.29^abc^ | 3.69^ab^ | 3.95^ab^ | 4.15^abc^ |  | 2 |  |  |  |  |  |  |  |  |
| Heart rate, beats/min |  | 114 | 120 | 111 | 113 | 3.2 |  | 119 | 116 | 112 | 110 | 3.1 | 115 | 114 | 2.3 |
| Heat energy, MJ/d |  | 15.95 | 17.78 | 14.51 | 16.41 | 0.832 |  | 16.87 | 16.24 | 15.89 | 15.64 | 0.563 | 15.84 | 16.49 | 0.588 |
|  | 1 | 16.66^bcde^ | 19.77^e^ | 14.53^ab^ | 16.53^bcd^ | 1.055 |  |  |  |  |  |  |  |  |  |
|  | 2 | 15.40^abc^ | 19.12^de^ | 14.78^abc^ | 15.68^bc^ |  |  |  |  |  |  |  |  |  |  |
|  | 3 | 15.86^bc^ | 16.69^bcde^ | 13.18^a^ | 17.83^cde^ |  |  |  |  |  |  |  |  |  |  |
|  | 4 | 15.88^bc^ | 15.51^abc^ | 15.57^bc^ | 15.60^bc^ |  |  |  |  |  |  |  |  |  |  |
| Heat energy, kJ/kg BW^0.75^ |  | 759 | 826 | 730 | 815 | 34.1 |  | 813 | 793 | 765 | 761 | 24.9 | 775 | 790 | 24.1 |
|  | 1 | 787^abcd^ | 917^d^ | 728^ab^ | 820^bcd^ | 49.8 |  |  |  |  |  |  |  |  |  |
|  | 2 | 740^bc^ | 895^d^ | 747^abc^ | 788^abcd^ |  |  |  |  |  |  |  |  |  |  |
|  | 3 | 755^abc^ | 763^abc^ | 667^a^ | 874^cd^ |  |  |  |  |  |  |  |  |  |  |
|  | 4 | 756^abc^ | 731^ab^ | 778^abcd^ | 778^abcd^ |  |  |  |  |  |  |  |  |  |  |
| Milk composition |  |  |  |  |  |  |  |  |  |  |  |  |  |  |  |
| Fat, % |  | 3.16^ab^ | 3.37^b^ | 2.93^a^ | 2.97^a^ | 0.120 |  | 3.36^b^ | 3.00^a^ | 2.99^a^ | 3.08^a^ | 0.071 | 3.14 | 3.07 | 0.085 |
| Protein, % |  | 2.62^ab^ | 2.69^b^ | 2.58^ab^ | 2.52^a^ | 0.042 |  | 2.81^c^ | 2.57^b^ | 2.53^ab^ | 2.51^a^ | 0.026 | 2.62 | 2.59 | 0.030 |
| Lactose, % |  | 4.43 | 4.43 | 4.35 | 4.35 | 0.034 |  | 4.55^c^ | 4.39^b^ | 4.36^b^ | 4.26^a^ | 0.019 | 4.40 | 4.38 | 0.024 |
|  | 1 | 4.60^fg^ | 4.61^g^ | 4.50^ef^ | 4.51^efg^ | 0.038 | 1 | 4.54^c^ | 4.41^b^ | 4.37^b^ | 4.26^a^ | 0.027 |  |  |  |
|  | 2 | 4.43^cde^ | 4.45^de^ | 4.33^ab^ | 4.34^abc^ |  | 2 | 4.57^c^ | 4.36^b^ | 4.35^b^ | 4.25^a^ |  |  |  |  |
|  | 3 | 4.37^bcd^ | 4.41^de^ | 4.32^ab^ | 4.32^ab^ |  |  |  |  |  |  |  |  |  |  |
|  | 4 | 4.31^a^ | 4.24^a^ | 4.24^a^ | 4.25^a^ |  |  |  |  |  |  |  |  |  |  |
| TS, % |  | 10.77^ab^ | 11.04^b^ | 10.41^a^ | 10.41^a^ | 0.159 |  | 11.31^b^ | 10.52^a^ | 10.43^a^ | 10.38^a^ | 0.091 | 10.72 | 10.60 | 0.112 |
| SNF, % |  | 7.61^ab^ | 7.66^b^ | 7.48^a^ | 7.44^a^ | 0.062 |  | 7.94^c^ | 7.50^b^ | 7.44^b^ | 7.30^a^ | 0.035 | 7.57 | 7.52 | 0.044 |
| Urea nitrogen, mg/dL |  | 21.4 | 21.9 | 21.9 | 21.1 | 0.79 |  | 20.7^a^ | 20.9^a^ | 23.3^b^ | 21.4^a^ | 0.50 | 21.2 | 22.0 | 0.56 |
| Log SCC, /mL |  | 6.37 | 6.63 | 6.47 | 6.61 | 0.273 |  | 6.50^b^ | 6.21^a^ | 6.53^b^ | 6.85^c^ | 0.152 | 6.60 | 6.44 | 0.191 |
| Milk yield |  |  |  |  |  |  |  |  |  |  |  |  |  |  |  |
| Raw, kg/d |  | 2.82^b^ | 2.71^b^ | 2.23^a^ | 2.10^a^ | 0.157 |  | 2.65^c^ | 2.55^b^ | 2.50^b^ | 2.18^a^ | 0.083 | 2.31^a^ | 2.63^b^ | 0.111 |
|  | 1 | 2.85^f^ | 2.87^f^ | 2.54^def^ | 2.32^cde^ | 0.169 | 1 | 2.36^bc^ | 2.37^bc^ | 2.39^bc^ | 2.11^a^ | 0.118 |  |  |  |
|  | 2 | 2.96^f^ | 2.90^f^ | 2.27^bc^ | 2.07^ab^ |  | 2 | 2.93^e^ | 2.73^d^ | 2.62^cd^ | 2.25^ab^ |  |  |  |  |
|  | 3 | 2.90^f^ | 2.78^ef^ | 2.22^bc^ | 2.10^ab^ |  |  |  |  |  |  |  |  |  |  |
|  | 4 | 2.61^def^ | 2.30^bcd^ | 1.88^a^ | 1.92^ab^ |  |  |  |  |  |  |  |  |  |  |
| Fat, g/d |  | 90.7^b^ | 92.3^b^ | 65.9^a^ | 62.7^a^ | 6.60 |  | 89.3^c^ | 77.4^b^ | 76.4^b^ | 68.5^a^ | 3.48 | 73.2 | 82.6 | 4.67 |
|  | 1 | 96.5^cd^ | 102.7^d^ | 79.8^c^ | 78.1^bc^ | 6.96 | 1 | 80.6^d^ | 71.9^bc^ | 73.4^bc^ | 66.9^a^ | 4.92 |  |  |  |
|  | 2 | 89.0^cd^ | 96.0^cd^ | 64.3^ab^ | 60.6^a^ |  | 2 | 97.9^e^ | 83.0^d^ | 70.4^cd^ | 70.1^ab^ |  |  |  |  |
|  | 3 | 92.7^cd^ | 92.8^cd^ | 62.7^ab^ | 57.4^a^ |  |  |  |  |  |  |  |  |  |  |
|  | 4 | 84.7^cd^ | 77.9^bc^ | 56.8^a^ | 54.8^a^ |  |  |  |  |  |  |  |  |  |  |
| Protein, g/d |  | 74.5^b^ | 73.5^b^ | 57.9^a^ | 52.8^a^ | 4.41 |  | 74.6^c^ | 66.0^b^ | 63.4^b^ | 54.7^a^ | 2.36 | 60.8 | 68.5 | 3.12 |
|  | 1 | 81.2^fg^ | 83.5^g^ | 70.3^cdef^ | 63.5^bcd^ | 4.72 | 1 | 66.6^de^ | 61.6^bc^ | 61.3^bc^ | 53.8^a^ | 3.33 |  |  |  |
|  | 2 | 77.6^fg^ | 77.5^efg^ | 58.2^ab^ | 50.8^a^ |  | 2 | 82.6^f^ | 70.4^e^ | 65.6^cd^ | 55.6^ab^ |  |  |  |  |
|  | 3 | 73.8^defg^ | 73.5^def^ | 56.2^ab^ | 50.3^a^ |  |  |  |  |  |  |  |  |  |  |
|  | 4 | 65.3^bcde^ | 59.7^abc^ | 47.1^a^ | 46.6^a^ |  |  |  |  |  |  |  |  |  |  |
| Energy, MJ/d |  | 7.51^b^ | 7.45^b^ | 5.68^a^ | 5.34^a^ | 0.471 |  | 7.30^c^ | 6.57^b^ | 6.43^b^ | 5.67^a^ | 0.247 | 6.09 | 6.89 | 0.333 |
|  | 1 | 7.90^fg^ | 8.21^g^ | 6.75^def^ | 6.35^cde^ | 0.494 | 1 | 6.55^bc^ | 6.11^bc^ | 6.17^bc^ | 5.52^a^ | 0.349 |  |  |  |
|  | 2 | 7.61^efg^ | 7.83^fg^ | 5.60^abc^ | 5.18^ab^ |  | 2 | 8.05^d^ | 7.03^c^ | 6.69^c^ | 5.81^ab^ |  |  |  |  |
|  | 3 | 7.63^efg^ | 7.52^efg^ | 5.50^abc^ | 5.07^ab^ |  |  |  |  |  |  |  |  |  |  |
|  | 4 | 6.89^defg^ | 6.24^bcd^ | 4.80^a^ | 4.74^a^ |  |  |  |  |  |  |  |  |  |  |
| Energy, MJ/kg DMI |  | 2.97 | 2.98 | 2.78 | 2.88 | 0.165 |  | 3.40^c^ | 2.88^b^ | 2.68^a^ | 2.65^a^ | 0.099 | 2.71^a^ | 3.09^b^ | 0.116 |
|  | 1 | 3.08^cd^ | 3.21^cde^ | 3.58^de^ | 3.75^e^ | 0.197 |  |  |  |  |  |  |  |  |  |
|  | 2 | 2.93^bc^ | 3.07^cd^ | 2.73^bc^ | 2.80^bc^ |  |  |  |  |  |  |  |  |  |  |
|  | 3 | 2.95^bc^ | 2.80^bc^ | 2.48^ab^ | 2.47^ab^ |  |  |  |  |  |  |  |  |  |  |
|  | 4 | 2.93^bc^ | 2.82^bc^ | 2.37^a^ | 2.50^ab^ |  |  |  |  |  |  |  |  |  |  |
| Lactose, g/d |  | 125.9^b^ | 120.2^b^ | 97.5^a^ | 91.7^a^ | 7.13 |  | 120.8^c^ | 112.4^b^ | 109.2^b^ | 93.0^a^ | 3.79 | 101.4^a^ | 116.3^b^ | 5.04 |
|  | 1 | 131.2^f^ | 132.3^f^ | 114.9^def^ | 104.9^cde^ | 7.57 | 1 | 107.0^bcd^ | 104.7^bc^ | 104.2^bc^ | 89.8^a^ | 5.36 |  |  |  |
|  | 2 | 131.8^f^ | 120.0^f^ | 98.9^bcd^ | 89.8^ab^ |  | 2 | 134.6^e^ | 120.0^d^ | 114.1^cd^ | 96.3^ab^ |  |  |  |  |
|  | 3 | 127.6^f^ | 122.4^ef^ | 96.2^bc^ | 90.6^ab^ |  |  |  |  |  |  |  |  |  |  |
|  | 4 | 113.1^cde^ | 97.3^bcd^ | 80.2^a^ | 81.5^ab^ |  |  |  |  |  |  |  |  |  |  |
| TS, g/d |  | 307.1^b^ | 301.0^b^ | 233.9^a^ | 219.1^a^ | 18.50 |  | 300.1^c^ | 270.2^b^ | 262.9^b^ | 227.9^a^ | 9.73 | 248.4 | 282.2 | 13.08 |
|  | 1 | 325.8^ef^ | 335.1^f^ | 279.6^cde^ | 259.8^bcd^ | 19.25 | 1 | 267.8^b^ | 251.7^b^ | 252.2^b^ | 221.7^a^ | 13.77 |  |  |  |
|  | 2 | 315.2^ef^ | 318.7^ef^ | 234.2^ab^ | 212.6^a^ |  | 2 | 332.4^b^ | 288.7^b^ | 273.6^b^ | 234.1^ab^ |  |  |  |  |
|  | 3 | 310.2^def^ | 303.8^de^ | 227.4^ab^ | 210.2^a^ |  |  |  |  |  |  |  |  |  |  |
|  | 4 | 277.3^bcd^ | 246.3^abc^ | 194.3^a^ | 193.6^a^ |  |  |  |  |  |  |  |  |  |  |
| SNF, g/d |  | 216.2^b^ | 208.4^b^ | 167.8^a^ | 156.4^a^ | 8.68 |  | 210.8^c^ | 192.0^b^ | 186.5^b^ | 159.3^a^ | 6.52 | 174.9 | 199.5 | 8.68 |
|  | 1 | 229.4^g^ | 232.4^g^ | 199.8^defg^ | 181.7^cde^ | 8.68 | 1 | 187.2^bcd^ | 178.9^bc^ | 178.8^bc^ | 154.8^a^ | 9.22 |  |  |  |
|  | 2 | 225.4^g^ | 221.5^fg^ | 169.0^bcd^ | 152.1^ab^ |  | 2 | 234.5^e^ | 205.1^d^ | 194.3^cd^ | 163.9^ab^ |  |  |  |  |
|  | 3 | 217.4^efg^ | 210.0^efg^ | 164.8^bc^ | 152.9^ab^ |  |  |  |  |  |  |  |  |  |  |
|  | 4 | 192.6^cdef^ | 168.5^bcd^ | 137.5^a^ | 138.8^ab^ |  |  |  |  |  |  |  |  |  |  |
| BW = body weight; DMI = dry matter intake; TS = total solids; SNF = solids-non-fat; SCC = somatic cell count.  ^1^Diets were 40%, 50%, 60%, and 70% forage (40F, 50F, 60F, and 70F, respectively), with forage in 60F and 70F diet being grass hay (primarily orchardgrass) and that in 40F and  50F cottonseed hulls, dehydrated alfalfa pellets, and wheat hay.  ^2^Periods (PRD) were 28 d in length.  ^3^Parities (PAR) were first vs. multiple lactations (1 and 2, respectively).  ^a–g^Means within grouping without a common superscript letter differ (*P* < 0.05). | | | | | | | | | | | | | | | |

| **Table S4**  *P-*values for effects of dietary treatment, parity, period, parity, and sampling time on ruminal fluid characteristics. | | | | | | | | | | |
| --- | --- | --- | --- | --- | --- | --- | --- | --- | --- | --- |
| Source of variation^1^ | Variable | | | | | | | | | |
|  | pH | VFA, mM | AC, % | PR, % | IB, % | BT, % | IV, % | VL, % | AC:PR | AMN, mg/dL |
| TRT | <0.001 | 0.156 | <0.001 | 0.091 | 0.008 | <0.001 | 0.005 | <0.001 | 0.434 | 0.990 |
| PAR | 0.845 | 0.833 | 0.118 | 0.762 | 0.178 | 0.230 | 0.758 | 0.065 | 0.716 | 0.841 |
| TRT × PAR | 0.731 | 0.752 | 0.810 | 0.961 | 0.496 | 0.753 | 0.794 | 0.904 | 0.947 | 0616 |
| PRD | <0.001 | <0.001 | <0.001 | <0.001 | <0.001 | <0.001 | <0.001 | <0.001 | <0.001 | 0.020 |
| TRT × PRD | 0.023 | 0.144 | 0.107 | 0.004 | 0.546 | 0.052 | 0.709 | 0.007 | 0.053 | <0.001 |
| PAR × PRD | 0.447 | 0.208 | 0.836 | 0.775 | 0.262 | 0.792 | 0.518 | 0.296 | 0.281 | 0.725 |
| TRT × PAR × PRD | 0.164 | 0.031 | 0.028 | 0.275 | 0.029 | 0.006 | 0.049 | 0.009 | 0.267 | 0.149 |
| TIME | <0.001 | <0.001 | 0.001 | <0.001 | <0.001 | 0.322 | <0.001 | <0.001 | <0.001 | <0.001 |
| TRT × TIME | 0.136 | 0.077 | 0.237 | <0.001 | 0.165 | 0.009 | 0.189 | 0.344 | <0.001 | 0.348 |
| PAR × TIME | 0.041 | 0.142 | 0.947 | 0.113 | 0.053 | 0.245 | 0.042 | 0.324 | 0.065 | 0.099 |
| TRT × PAR × TIME | 0.884 | 0.981 | 0.518 | 0.047 | 0.440 | 0.739 | 0.264 | 0.311 | 0.082 | 0.027 |
| PRD × TIME | <0.001 | <0.001 | 0.032 | 0.041 | 0.028 | 0.248 | 0.158 | 0.970 | 0.005 | 0.103 |
| TRT × PRD × TIME | 0.845 | 0.125 | 0.259 | 0.151 | 0.536 | 0.483 | 0.468 | 0.209 | 0.100 | 0.372 |
| PAR × PRD × TIME | 0.320 | 0.145 | 0.751 | 0.598 | 0.171 | 0.829 | 0.321 | 0.917 | 0.524 | 0.876 |
| TRT × PAR × PRD × TIME | 0.964 | 0.311 | 0.060 | 0.559 | 0.360 | 0.281 | 0.308 | 0.106 | 0.598 | 0.525 |
| VFA = volatile fatty acids; AC = acetate; PR = propionate; IB = isobutyrate; BT = butyrate; IV = isovalerate; AMN =  ammonia nitrogen.  ^1^TRT = dietary treatment; PAR = parity (first vs. multiple lactations); PRD = period; TIME = sampling time. | | | | | | | | | | |

| **Table S5**  Effects of dietary treatment, period, and sampling time on ruminal fluid pH and acetate:propionate ratio to address two-way interactions. | | | | | | | | | | | | | | | | | |
| --- | --- | --- | --- | --- | --- | --- | --- | --- | --- | --- | --- | --- | --- | --- | --- | --- | --- |
| Item^1^ | PRD | TIME | PAR^2^ |  | Diet^3^ | | | | SEM | Period^4^ | | | | SEM | Time^5^ | | SEM |
|  |  |  |  |  | 40F | 50F | 60F | 70F |  | 1 | 2 | 3 | 4 |  | AM | PM |  |
| pH | 1 |  |  |  | 5.92^def^ | 5.82^cd^ | 6.01^fg^ | 6.11^g^ | 0.043 |  |  |  |  |  |  |  |  |
|  | 2 |  |  |  | 5.71^abc^ | 5.81^bcd^ | 5.91^de^ | 5.97^ef^ |  |  |  |  |  |  |  |  |  |
|  | 3 |  |  |  | 5.68^a^ | 5.60^a^ | 5.85^de^ | 5.93^def^ |  |  |  |  |  |  |  |  |  |
|  | 4 |  |  |  | 5.70^ab^ | 5.63^a^ | 5.87^de^ | 5.88^de^ |  |  |  |  |  |  |  |  |  |
|  |  | AM |  |  |  |  |  |  |  | 6.07^f^ | 5.89^cd^ | 5.94^de^ | 5.96^e^ | 0.026 |  |  |  |
|  |  | PM |  |  |  |  |  |  |  | 5.86^bc^ | 5.82^b^ | 5.60^a^ | 5.58^a^ |  |  |  |  |
|  |  |  | 1 |  |  |  |  |  |  |  |  |  |  |  | 5.94^b^ | 5.73^a^ | 0.027 |
|  |  |  | 2 |  |  |  |  |  |  |  |  |  |  |  | 5.98^b^ | 5.70^a^ |  |
| AC:PR |  | AM |  |  | 4.84^c^ | 4.87^c^ | 4.42^b^ | 4.68^bc^ | 0.120 | 4.36^bc^ | 4.32^b^ | 4.72^cd^ | 5.39^e^ | 0.091 |  |  |  |
|  |  | PM |  |  | 3.91^a^ | 4.15^ab^ | 4.21^ab^ | 4.37^b^ |  | 3.95^a^ | 3.99^a^ | 4.16^ab^ | 4.54^cd^ |  |  |  |  |
| ^1^AC:PR = acetate:propionate ratio.  ^2^PAR = parities were first vs. multiple lactations (1 and 2, respectively).  ^3^Diets were 40%, 50%, 60%, and 70% forage (40F, 50F, 60F, and 70F, respectively), with forage in 60F and 70F diet being grass hay (primarily  orchardgrass) and that in 40F and 50F cottonseed hulls, dehydrated alfalfa pellets, and wheat hay.  ^4^Periods were 28 d in length.  ^5^AM samples were collected at 3 to 4 h after the feeding in the morning and PM samples were before the afternoon milking about 7 to 8 h after feeding.  ^a–g^Means within grouping without a common superscript letter differ (*P* < 0.05). | | | | | | | | | | | | | | | | | |

| **Table S6**  *P*-values for effects of dietary treatment, period, and sampling time on ruminal fluid characteristics for doelings in the first lactation and does. | | | | | | | | |
| --- | --- | --- | --- | --- | --- | --- | --- | --- |
| PAR^1^ | Item | Source of variation^2^ | | | | | | |
|  |  | TRT | PRD | TRT × PRD | TIME | TRT × TIME | PRD × TIME | TRT × PRD × TIME |
| 1 | VFA, mM | 0.217 | <0.001 | 0.033 | 0.068 | 0.273 | <0.001 | 0.293 |
|  | Acetate, % | <0.001 | <0.001 | 0.008 | 0.003 | 0.549 | 0.361 | 0.144 |
|  | Propionate, % | 0.309 | <0.001 | 0.389 | <0.001 | 0.105 | 0.337 | 0.927 |
|  | Isobutyrate, % | 0.006 | <0.001 | 0.022 | <0.01 | 0.104 | <0.001 | 0.390 |
|  | Butyrate, % | 0.002 | <0.001 | <0.001 | 0.034 | 0.172 | 0.011 | 0.781 |
|  | Isovalerate, % | 0.007 | <0.001 | 0.120 | <0.001 | 0.121 | 0.001 | 0.656 |
|  | Valerate, % | 0.002 | <0.001 | <0.001 | <0.001 | 0.247 | 0.175 | 0.306 |
|  | AMN, mg/dL | 0.682 | 0.051 | 0.001 | 0.008 | 0.057 | 0.272 | 0.271 |
| 2 | VFA, mM | 0.437 | <0.001 | 0.085 | <0.001 | 0.298 | 0.032 | 0.184 |
|  | Acetate, % | 0.001 | <0.001 | 0.175 | 0.049 | 0.297 | 0.123 | 0.135 |
|  | Propionate, % | 0.317 | <0.001 | 0.007 | <0.001 | <0.001 | 0.103 | 0.081 |
|  | Isobutyrate, % | 0.247 | <0.001 | 0.957 | <0.001 | 0.261 | 0.001 | 0.249 |
|  | Butyrate, % | 0.002 | <0.001 | 0.006 | 0.004 | 0.115 | 0.028 | 0.033 |
|  | Isovalerate, % | 0.129 | <0.001 | 0.942 | <0.001 | 0.186 | 0.003 | 0.269 |
|  | Valerate, % | 0.021 | <0.001 | 0.184 | <0.001 | 0.592 | 0.134 | 0.028 |
|  | AMN, mg/dL | 0.908 | 0.331 | 0.049 | <0.001 | 0.215 | 0.399 | 0.692 |
| VFA = volatile fatty acids; AMN = ammonia nitrogen.  ^1^PAR = parities were first vs. multiple lactations (1 and 2, respectively).  ^2^TRT = dietary treatment; PRD = period; TIME = sampling time. | | | | | | | | |

| **Table S7**  Effects of dietary treatment, period, and sampling time on ruminal fluid characteristics of Alpine doelings in the first lactation to address three-way interactions involving parity. | | | | | | | | | | | | | | | | |
| --- | --- | --- | --- | --- | --- | --- | --- | --- | --- | --- | --- | --- | --- | --- | --- | --- |
| Item | Period | Time |  | Diet^1^ | | | | SEM | Period^2^ | | | | SEM | Time^3^ | | SEM |
|  |  |  |  | 40F | 50F | 60F | 70F |  | 1 | 2 | 3 | 4 |  | AM | PM |  |
| VFA, mM | 1 |  |  | 72.9^e^ | 83.2^f^ | 67.8^cde^ | 67.2^cde^ | 3.34 |  |  |  |  |  |  |  |  |
|  | 2 |  |  | 64.5^bcd^ | 72.9^e^ | 67.4^cde^ | 70.1^de^ |  |  |  |  |  |  |  |  |  |
|  | 3 |  |  | 63.2^bcd^ | 63.9^bcd^ | 65.1^bcde^ | 60.1^abc^ |  |  |  |  |  |  |  |  |  |
|  | 4 |  |  | 55.0^a^ | 53.4^a^ | 51.7^a^ | 56.8^ab^ |  |  |  |  |  |  |  |  |  |
|  |  | AM |  |  |  |  |  |  | 72.8^d^ | 71.1^cd^ | 61.9^b^ | 47.4^a^ | 2.25 |  |  |  |
|  |  | PM |  |  |  |  |  |  | 72.7^d^ | 66.3^bc^ | 64.3^b^ | 61.0^b^ |  |  |  |  |
| AC, % | 1 |  |  | 65.2^ab^ | 64.6^a^ | 68.7^cd^ | 72.4^fg^ | 1.02 |  |  |  |  |  |  |  |  |
|  | 2 |  |  | 65.3^ab^ | 66.8^bc^ | 70.0^defg^ | 72.1^efg^ |  |  |  |  |  |  |  |  |  |
|  | 3 |  |  | 68.5^cd^ | 67.2^bc^ | 70.6^defg^ | 71.2^defg^ |  |  |  |  |  |  |  |  |  |
|  | 4 |  |  | 69.3^cde^ | 71.1^defg^ | 72.5^gh^ | 74.8^h^ |  |  |  |  |  |  |  |  |  |
| PR, % |  |  |  | 15.9 | 15.3 | 16.4 | 16.2 | 0.47 | 16.5^c^ | 16.9^c^ | 15.6^b^ | 14.8^a^ | 0.31 | 69.8^b^ | 68.9^a^ | 0.47 |
| IB, % | 1 |  |  | 0.63^g^ | 0.65^g^ | 0.53^ef^ | 0.43^bcd^ | 0.029 |  |  |  |  |  |  |  |  |
|  | 2 |  |  | 0.51^def^ | 0.53^f^ | 0.45^cde^ | 0.44^bcde^ |  |  |  |  |  |  |  |  |  |
|  | 3 |  |  | 0.47^cdef^ | 0.45^cde^ | 0.45^cdef^ | 0.42^bc^ |  |  |  |  |  |  |  |  |  |
|  | 4 |  |  | 0.36^ab^ | 0.36^ab^ | 0.32^a^ | 0.31^a^ |  |  |  |  |  |  |  |  |  |
|  |  | AM |  |  |  |  |  |  | 0.64^f^ | 0.57^e^ | 0.56^e^ | 0.37^bc^ | 0.020 |  |  |  |
|  |  | PM |  |  |  |  |  |  | 0.47^d^ | 0.39^c^ | 0.33^ab^ | 0.30^a^ |  |  |  |  |
| BT, % | 1 |  |  | 10.8^e^ | 14.1^f^ | 7.7^bcde^ | 5.8^abc^ | 1.05 |  |  |  |  |  |  |  |  |
|  | 2 |  |  | 9.4^de^ | 10.9^e^ | 6.6^abc^ | 5.5^ab^ |  |  |  |  |  |  |  |  |  |
|  | 3 |  |  | 8.6^cd^ | 9.4d^e^ | 6.8^abcd^ | 6.2^abc^ |  |  |  |  |  |  |  |  |  |
|  | 4 |  |  | 7.3^abcde^ | 6.9^abcd^ | 5.5^ab^ | 4.7^a^ |  |  |  |  |  |  |  |  |  |
|  |  | AM |  |  |  |  |  |  | 9.5^c^ | 8.1^b^ | 7.9^b^ | 5.0^a^ | 0.56 |  |  |  |
|  |  | PM |  |  |  |  |  |  | 9.7^c^ | 8.1^b^ | 7.8^b^ | 7.1^b^ |  |  |  |  |
| IV, % |  |  |  | 0.73^c^ | 0.73^c^ | 0.64^ab^ | 0.57^a^ | 0.034 |  |  |  |  |  |  |  |  |
|  |  | AM |  |  |  |  |  |  | 0.99^d^ | 0.88^c^ | 0.89^c^ | 0.61^b^ | 0.037 |  |  |  |
|  |  | PM |  |  |  |  |  |  | 0.62^b^ | 0.50^a^ | 0.43^a^ | 0.42^a^ |  |  |  |  |
| VL, % | 1 |  |  | 0.90^e^ | 1.11^f^ | 0.69^cd^ | 0.53^abc^ | 0.059 |  |  |  |  |  |  |  |  |
|  | 2 |  |  | 0.77^d^ | 0.82^de^ | 0.63^c^ | 0.56^abc^ |  |  |  |  |  |  |  |  |  |
|  | 3 |  |  | 0.67^c^ | 0.73^d^ | 0.61^abc^ | 0.62^bc^ |  |  |  |  |  |  |  |  |  |
|  | 4 |  |  | 0.56^abc^ | 0.55^abc^ | 0.45^ab^ | 0.44^a^ |  |  |  |  |  |  |  |  |  |
|  |  |  |  |  |  |  |  |  |  |  |  |  |  | 0.62^a^ | 0.72^b^ | 0.026 |
| AMN, mg/dL | 1 |  |  | 24.6^bcde^ | 22.9^abcd^ | 26.3^de^ | 18.5^a^ | 1.64 |  |  |  |  |  |  |  |  |
|  | 2 |  |  | 27.3^e^ | 22.7^abcd^ | 22.0^abcd^ | 23.0^bcde^ |  |  |  |  |  |  |  |  |  |
|  | 3 |  |  | 21.9^abcd^ | 21.3^ab^ | 21.5^abc^ | 21.3^ab^ |  |  |  |  |  |  |  |  |  |
|  | 4 |  |  | 22.2^abcd^ | 22.4^abcd^ | 23.3^bcde^ | 26.1^cde^ |  |  |  |  |  |  |  |  |  |
|  |  |  |  |  |  |  |  |  |  |  |  |  |  | 23.8^b^ | 22.1^a^ | 0.70 |
| VFA = volatile fatty acids; AC = acetate; PR = propionate; IB = isobutyrate; BT = butyrate; IV = isovalerate; VL = valerate; AMN = ammonia nitrogen.  ^1^Diets were 40%, 50%, 60%, and 70% forage (40F, 50F, 60F, and 70F, respectively), with forage in 60F and 70F diet being grass hay (primarily orchardgrass) and that in 40F and 50F cottonseed hulls, dehydrated alfalfa pellets, and wheat hay.  ^2^Periods were 28 d in length.  ^3^AM samples were collected at 3 to 4 h after the feeding in the morning and PM samples were at 7 to 8 h after feeding.  ^a–h^Means within grouping without a common superscript letter differ (*P* < 0.05). | | | | | | | | | | | | | | | | |

| **Table S8**  Effects of dietary treatment, period, and sampling time on ruminal fluid characteristics of Alpine does to address three-way interactions involving parity. | | | | | | | | | | | | | | | | |
| --- | --- | --- | --- | --- | --- | --- | --- | --- | --- | --- | --- | --- | --- | --- | --- | --- |
| Item | Period | Time |  | Diet^1^ | | | | SEM | Period^2^ | | | | SEM | Time^3^ | | SEM |
|  |  |  |  | 40F | 50F | 60F | 70F |  | 1 | 2 | 3 | 4 |  | AM | PM |  |
| VFA, mM |  |  |  | 65.7 | 66.3 | 63.8 | 61.8 | 2.12 |  |  |  |  |  |  |  |  |
|  |  | AM |  |  |  |  |  |  | 66.9^c^ | 66.4^c^ | 66.7^c^ | 45.6^a^ | 2.33 |  |  |  |
|  |  | PM |  |  |  |  |  |  | 74.2^d^ | 70.6^cd^ | 66.4^c^ | 58.2^b^ |  |  |  |  |
| AC, % |  |  |  | 65.4^a^ | 67.2^ab^ | 69.4^bc^ | 71.2^c^ | 0.96 | 66.5^a^ | 67.5^ab^ | 68.2^b^ | 71.0^c^ | 0.61 | 68.7^b^ | 67.9^a^ | 0.53 |
| PR, % | 1 |  |  | 15.2^bc^ | 15.9^abcde^ | 17.8^ef^ | 17.2^def^ | 0.69 |  |  |  |  |  |  |  |  |
|  | 2 |  |  | 16.7^bcdef^ | 15.4^abcd^ | 18.5^f^ | 16.8^cdef^ |  |  |  |  |  |  |  |  |  |
|  | 3 |  |  | 15.5^bcd^ | 16.1^abcde^ | 16.1^abcde^ | 16.4^bcde^ |  |  |  |  |  |  |  |  |  |
|  | 4 |  |  | 15.6^abcd^ | 14.7^ab^ | 14.5^a^ | 14.3^a^ |  |  |  |  |  |  |  |  |  |
|  |  | AM |  | 13.9^a^ | 14.2^ab^ | 16.7^c^ | 15.7^b^ | 0.56 |  |  |  |  |  |  |  |  |
|  |  | PM |  | 17.6^c^ | 16.8^c^ | 16.7^c^ | 16.7^c^ |  |  |  |  |  |  |  |  |  |
| IB, % |  |  |  | 0.50 | 0.49 | 0.46 | 0.44 | 0.023 |  |  |  |  |  |  |  |  |
|  |  | AM |  |  |  |  |  |  | 0.70^e^ | 0.57^d^ | 0.62^d^ | 0.35^b^ | 0.024 |  |  |  |
|  |  | PM |  |  |  |  |  |  | 0.48^c^ | 0.40^b^ | 0.36^b^ | 0.27^a^ |  |  |  |  |
| BT^4^, % | 1 | AM |  | 11.7^cd^ | 11.5^cd^ | 7.6^ab^ | 6.2^a^ | 1.17 |  |  |  |  |  |  |  |  |
|  |  | PM |  | 13.5^d^ | 10.2^bcd^ | 9.7^bc^ | 7.5^ab^ |  |  |  |  |  |  |  |  |  |
|  | 2 |  |  | 11.7^b^ | 9.0^a^ | 7.0^a^ | 7.1^a^ | 0.81 |  |  |  |  |  | 8.5 | 8.9 | 0.45 |
|  | 3 | AM |  | 11.2^c^ | 11.8^c^ | 6.7^ab^ | 6.0^a^ | 1.28 |  |  |  |  |  |  |  |  |
|  |  | PM |  | 9.0^abc^ | 10.0^bc^ | 8.8^abc^ | 6.8^ab^ |  |  |  |  |  |  |  |  |  |
|  | 4 |  |  | 7.1^bc^ | 7.6^c^ | 5.4^ab^ | 4.4^a^ | 0.68 |  |  |  |  |  | 5.2^a^ | 7.0^b^ | 0.41 |
| IV, % |  |  |  | 0.73 | 0.71 | 0.64 | 0.61 | 0.041 |  |  |  |  |  |  |  |  |
|  |  | AM |  |  |  |  |  |  | 1.07^e^ | 0.86^d^ | 0.98^e^ | 0.58^c^ | 0.042 |  |  |  |
|  |  | PM |  |  |  |  |  |  | 0.59^c^ | 0.46^b^ | 0.49^bc^ | 0.35^a^ |  |  |  |  |
| VL^4^, % | 1 |  |  | 0.98 | 0.95 | 0.83 | 0.66 | 0.093 |  |  |  |  |  | 0.76^a^ | 0.96^b^ | 0.051 |
|  | 2 |  |  | 0.93^b^ | 0.80^ab^ | 0.62^a^ | 0.69^a^ | 0.066 |  |  |  |  |  | 0.85^a^ | 1.02^b^ | 0.081 |
|  | 3 |  |  | 0.81 | 0.87 | 0.67 | 0.65 | 0.076 |  |  |  |  |  | 0.72 | 0.78 | 0.047 |
|  | 4 | AM |  | 0.35^a^ | 0.51^ab^ | 0.37^a^ | 0.36^a^ | 0.072 |  |  |  |  |  |  |  |  |
|  |  | PM |  | 0.82^c^ | 0.70^bc^ | 0.50^ab^ | 0.42^a^ |  |  |  |  |  |  |  |  |  |
| AMN, mg/dL | 1 |  |  | 25.4^b^ | 23.0^ab^ | 22.4^ab^ | 21.1^ab^ | 1.68 |  |  |  |  |  |  |  |  |
|  | 2 |  |  | 23.3^ab^ | 22.9^ab^ | 22.7^ab^ | 24.1^ab^ |  |  |  |  |  |  |  |  |  |
|  | 3 |  |  | 20.5^a^ | 24.0^ab^ | 21.2^ab^ | 23.7^ab^ |  |  |  |  |  |  |  |  |  |
|  | 4 |  |  | 20.8^a^ | 25.6^b^ | 24.9^ab^ | 24.8^ab^ |  |  |  |  |  |  |  |  |  |
|  |  |  |  |  |  |  |  |  |  |  |  |  |  | 24.7^b^ | 21.6^a^ | 0.77 |
| VFA = volatile fatty acids; AC = acetate; PR = propionate; IB = isobutyrate; BT = butyrate; IV = isovalerate; VL = valerate; AMN = ammonia nitrogen.  ^1^Diets were 40%, 50%, 60%, and 70% forage (40F, 50F, 60F, and 70F, respectively), with forage in 60F and 70F diet being grass hay (primarily  orchardgrass) and that in 40F and 50F cottonseed hulls, dehydrated alfalfa pellets, and wheat hay.  ^2^Periods were 28 d in length.  ^3^AM samples were collected at 3 to 4 h after the feeding in the morning and PM samples were at 7 to 8 h after feeding.  ^4^Analysis by period because of a significant three-way interaction between dietary treatment, period, and sampling time.  ^a–f^Means within grouping without a common superscript letter differ (*P* < 0.05). | | | | | | | | | | | | | | | | |

| **Table S9**  *P*-values for effects of dietary treatment, parity, period, parity, sampling time on blood constituent levels. | | | | | | | |
| --- | --- | --- | --- | --- | --- | --- | --- |
| Item | Source of variation^1^ | | | | | | |
|  | TRT | PAR | TRT × PAR | PRD | TRT × PRD | PAR × PRD | TRT × PAR × PRD |
| Total protein, g/dL | 0.554 | 0.411 | 0.429 | <0.001 | 0.146 | 0.058 | 0.574 |
| Albumin, g/dL | 0.251 | 0.891 | 0.740 | 0.520 | 0.221 | 0.612 | 0.986 |
| Cholesterol, mg/dL | 0.001 | 0.054 | 0.534 | <0.001 | 0.001 | 0.433 | 0.881 |
| Triglycerides, mg/dL | 0.727 | 0.870 | 0.285 | 0.001 | 0.083 | 0.207 | 0.994 |
| Glucose, mg/dL | 0.160 | 0.192 | 0.336 | <0.001 | 0.405 | 0.533 | 0.122 |
| Lactose, mg/dL | 0.032 | <0.001 | 0.224 | <0.001 | 0.152 | 0.551 | 0.558 |
| ^1^TRT = dietary treatment; PAR = parity (first vs. multiple lactations); PRD = period. | | | | | | | |

| **Table S10**  Effects of dietary treatment, period, and sampling time on blood constituent concentrations. | | | | | | | | | | | | | | | |
| --- | --- | --- | --- | --- | --- | --- | --- | --- | --- | --- | --- | --- | --- | --- | --- |
| Item | Period |  | Diet^1^ | | | | SEM | Period^2^ | | | | SEM | Parity^3^ | | SEM |
|  |  |  | 40F | 50F | 60F | 70F |  | 1 | 2 | 3 | 4 |  | 1 | 2 |  |
| Total protein, g/dL |  |  | 8.07 | 7.91 | 7.86 | 7.80 | 0.133 | 7.85^ab^ | 7.97^bc^ | 7.71^a^ | 8.09^c^ | 0.084 | 7.85 | 7.97 | 0.098 |
| Albumin, g/dL |  |  | 2.96 | 2.84 | 2.86 | 2.90 | 0.047 | 2.87 | 2.88 | 2.89 | 2.91 | 0.029 | 2.89 | 2.89 | 0.033 |
| Cholesterol, mg/dL |  |  | 139^c^ | 134^bc^ | 121^ab^ | 105^a^ | 6.0 | 115^a^ | 127^b^ | 126^b^ | 130^b^ | 3.4 | 119 | 131 | 4.3 |
|  | 1 |  | 126^bcd^ | 118^b^ | 120^bcd^ | 106^a^ | 6.7 |  |  |  |  |  |  |  |  |
|  | 2 |  | 138^cde^ | 135^cde^ | 121^bcd^ | 116^b^ |  |  |  |  |  |  |  |  |  |
|  | 3 |  | 140^e^ | 139^de^ | 122^bcd^ | 103^a^ |  |  |  |  |  |  |  |  |  |
|  | 4 |  | 150^e^ | 144^e^ | 120^bc^ | 105^b^ |  |  |  |  |  |  |  |  |  |
| Triglycerides, mg/dL |  |  | 33.4 | 33.1 | 31.8 | 32.1 | 1.19 | 29.8^a^ | 33.3^b^ | 34.4^b^ | 32.9^b^ | 0.93 | 32.7 | 32.5 | 0.84 |
| Glucose, mg/dL |  |  | 56.1 | 57.8 | 54.9 | 55.0 | 0.98 | 53.4^a^ | 56.9^bc^ | 58.0^c^ | 55.5^b^ | 0.75 | 56.6 | 55.3 | 0.69 |
| Lactate, mg/dL |  |  | 4.79^b^ | 4.80^b^ | 4.00^a^ | 4.14^ab^ | 0.240 | 4.01^a^ | 4.18^a^ | 4.86^b^ | 4.68^b^ | 0.172 | 4.92^b^ | 3.94^a^ | 0.170 |
| ^1^Diets were 40%, 50%, 60%, and 70% forage (40F, 50F, 60F, and 70F, respectively), with forage in 60F and 70F diet being grass hay (primarily orchardgrass) and that in 40F and 50F cottonseed hulls, dehydrated alfalfa pellets, and wheat hay.  ^2^Periods were 28 d in length and samples were collected at 3 to 4 h after feeding in the morning.  ^3^Parities were first vs. multiple lactations (1 and 2, respectively).  ^a–e^Means within grouping without a common superscript letter differ (*P* < 0.05). | | | | | | | | | | | | | | | |

| **Table S11**  *P*-values for effects of dietary treatment, parity, and period on body condition score, linear measures, and body mass indexes. | | | | | | | |
| --- | --- | --- | --- | --- | --- | --- | --- |
| Item^1^ | Source of variation^2^ | | | | | | |
|  | TRT | PAR | TRT × PAR | PRD | TRT × PRD | PAR × PRD | TRT × PAR × PRD |
| BCS | <0.001 | 0.389 | 0.667 | <0.001 | 0.537 | 0.251 | 0.878 |
| Hook, cm | 0.346 | 0.760 | 0.426 | 0.183 | 0.557 | 0.822 | 0.288 |
| Pin, cm | 0.184 | 0.957 | 0.299 | <0.001 | 0.117 | 0.069 | 0.386 |
| Heart girth, cm | 0.030 | 0.713 | 0.362 | 0.039 | 0.373 | 0.562 | 0.487 |
| Wither, cm | 0.876 | 0.052 | 0.874 | <0.001 | 0.117 | 0.329 | 0.841 |
| Rump, cm | 0.064 | 0.831 | 0.366 | <0.001 | 0.686 | 0.894 | 0.432 |
| BMI-WH, g/cm^2^ | 0.003 | 0.609 | 0.455 | <0.001 | 0.669 | 0.145 | 0.474 |
| BMI-WP, g/cm^2^ | 0.012 | 0.717 | 0.281 | <0.001 | 0.637 | 0.649 | 0.707 |
| BMI-GH, g/cm^2^ | 0.005 | 0.124 | 0.589 | <0.001 | 0.834 | 0.137 | 0.476 |
| BMI-GP, g/cm^2^ | 0.032 | 0.158 | 0.502 | <0.001 | 0.977 | 0.519 | 0.712 |
| ^1^BCS = body condition score (1–5); BMI = body mass index; Wither = height at withers; Hook = point of the shoulder to hook bone; Pin = point of the shoulder to pin bone; Rump = width at hook bones; BMI-WH = BW/(Wither × Hook); BMI-WP = BW/(Wither × Pin); BMI-GH = BW/(Heart girth × Hook); BMI-GP = BW/(Heart girth × Pin).  ^2^TRT = dietary treatment; PAR = parity (first vs. multiple lactations); PRD = period.  . | | | | | | | |

| **Table S12**  *P*-values for effects of dietary treatment, parity, and period on ingestive behavior and position on a daily basis. | | | | | | | |
| --- | --- | --- | --- | --- | --- | --- | --- |
| Item | Source of variation^1^ | | | | | | |
|  | TRT | PAR | TRT × PAR | PRD | TRT × PRD | PAR × PRD | TRT × PAR × PRD |
| Ingestive behavior |  |  |  |  |  |  |  |
| % day |  |  |  |  |  |  |  |
| Rumination | 0.854 | 0.718 | 0.015 | 0.389 | 0.085 | 0.165 | 0.355 |
| Eating | 0.836 | 0.268 | 0.139 | <0.001 | 0.261 | 0.287 | 0.001 |
| Idle | 0.728 | 0.342 | 0.064 | 0.226 | 0.275 | 0.516 | 0.073 |
| g DMI/min |  |  |  |  |  |  |  |
| Rumination | 0.124 | 0.475 | 0.186 | 0.597 | 0.438 | 0.688 | 0.231 |
| Eating | 0.099 | 0.621 | 0.104 | 0.002 | 0.616 | 0.312 | 0.031 |
| Position |  |  |  |  |  |  |  |
| Standing, % day | 0.729 | 0.209 | 0.505 | <0.001 | 0.936 | 0.711 | 0.947 |
| Lying, % day | 0.729 | 0.209 | 0.505 | <0.001 | 0.936 | 0.711 | 0.947 |
| Lying, right side |  |  |  |  |  |  |  |
| min/day | 0.690 | 0.744 | 0.750 | 0.009 | 0.816 | 0.255 | 0.221 |
| Lying, left side |  |  |  |  |  |  |  |
| min/day | 0.888 | 0.746 | 0.770 | 0.055 | 0.845 | 0.117 | 0.324 |
| DMI = dry matter intake.  ^1^TRT = dietary treatment; PAR = parity (first vs. multiple lactations); PRD = period. | | | | | | | |

| **Table S13**  Effects of dietary treatment, parity, and period on ingestive behavior and position on a daily basis. | | | | | | | | | | | | | | |
| --- | --- | --- | --- | --- | --- | --- | --- | --- | --- | --- | --- | --- | --- | --- |
| Item | Period | Dietary treatment^1^ | | | | SEM | Period^2^ | | | | SEM | Parity^3^ | | SEM |
|  |  | 40F | 50F | 60F | 70F |  | 1 | 2 | 3 | 4 |  | 1 | 2 |  |
| Ingestive behavior |  |  |  |  |  |  |  |  |  |  |  |  |  |  |
| % day |  |  |  |  |  |  |  |  |  |  |  |  |  |  |
| Rumination |  | 24.0 | 21.7 | 21.9 | 23.2 | 2.21 |  | 23.7 |  | 21.8 | 1.56 | 23.1 | 22.3 | 1.56 |
| Parity 1 |  | 21.9^abc^ | 28.4^c^ | 19.5^ab^ | 22.6^abc^ | 3.11 |  |  |  |  |  |  |  |  |
| Parity 2 |  | 26.2^bc^ | 15.0^a^ | 24.3^bc^ | 23.7^abc^ |  |  |  |  |  |  |  |  |  |
| Eating |  | 22.7 | 21.1 | 22.6 | 23.0 | 1.58 |  | 19.8^a^ |  | 24.9^b^ | 1.01 | 23.2 | 21.5 | 1.12 |
| Parity 1 | 2 | 16.9^a^ | 22.3^abcd^ | 18.7^abc^ | 22.1^abcd^ | 2.84 |  |  |  |  |  |  |  |  |
|  | 4 | 35.0^e^ | 19.3^abc^ | 23.6^abcd^ | 28.0^de^ |  |  |  |  |  |  |  |  |  |
| Parity 2 | 2 | 21.0^abcd^ | 18.8^abc^ | 21.7^abcd^ | 16.9^a^ |  |  |  |  |  |  |  |  |  |
|  | 4 | 17.9^ab^ | 24.0^abcd^ | 26.5^cd^ | 25.0^bcd^ |  |  |  |  |  |  |  |  |  |
| Idle |  | 53.3 | 57.2 | 55.5 | 53.9 | 2.66 |  | 56.6 |  | 53.4 | 1.88 | 53.7 | 56.3 | 1.88 |
| g DMI/min |  |  |  |  |  |  |  |  |  |  |  |  |  |  |
| Rumination |  | 8.48 | 12.33 | 8.26 | 8.51 | 1.389 |  | 9.03 |  | 9.77 | 0.983 | 8.90 | 9.90 | 0.983 |
| Eating |  |  |  |  |  |  |  |  |  |  |  |  |  |  |
| Parity 1 | 2 | 11.34^d^ | 10.34^cd^ | 7.78^abcd^ | 5.96^ab^ | 1.210 |  |  |  |  |  |  |  |  |
|  | 4 | 5.48^a^ | 10.00^cd^ | 6.72^ab^ | 4.54^a^ |  |  |  |  |  |  |  |  |  |
| Parity 2 | 2 | 9.37^bcd^ | 8.60^abcd^ | 7.57^abc^ | 9.41^bcd^ |  |  |  |  |  |  |  |  |  |
|  | 4 | 9.79^bcd^ | 6.96^abc^ | 6.13^ab^ | 7.50^abc^ |  |  |  |  |  |  |  |  |  |
| Position, % day |  |  |  |  |  |  |  |  |  |  |  |  |  |  |
| Standing |  | 29.6 | 29.4 | 28.3 | 32.6 | 2.79 | 38.6^b^ | 24.6^a^ | 28.0^a^ | 28.8^a^ | 2.26 | 31.8 | 28.2 | 1.97 |
| Lying |  | 70.4 | 70.6 | 71.7 | 67.3 | 2.79 | 61.4^a^ | 75.4^b^ | 72.0^b^ | 71.2^b^ | 2.26 | 68.2 | 71.8 | 1.97 |
| DMI = dry matter intake.  ^1^Diets were 40%, 50%, 60%, and 70% forage (40F, 50F, 60F, and 70F, respectively), with forage in 60F and 70F diet being grass hay (primarily orchardgrass) and that in 40F and 50F cottonseed hulls, dehydrated alfalfa pellets, and wheat hay.  ^2^Periods were 28 d in length.  ^3^Parities were first vs. multiple lactations (1 and 2, respectively).  ^a–e^Means within grouping without a common superscript letter differ (*P* < 0.05). | | | | | | | | | | | | | | |

| **Table S14**  *P*-values for effects of dietary treatment, parity, period, and hour of the day on ingestive behavior and position. | | | | | | |
| --- | --- | --- | --- | --- | --- | --- |
| Source of variation^1^ | Variable | | | | | |
|  | Ruminating | Eating | Idle | Standing | Lying, right side | Lying, left side |
| TRT | 0.787 | 0.749 | 0.659 | 0.768 | 0.923 | 0.967 |
| PAR | 0.962 | 0.146 | 0.413 | 0.258 | 0.666 | 0.612 |
| TRT × PAR | 0.025 | 0.229 | 0.128 | 0.540 | 0.926 | 0.751 |
| PRD | 0.498 | 0.001 | 0.265 | <0.001 | 0.027 | 0.041 |
| TRT × PRD | 0.097 | 0.261 | 0.294 | 0.873 | 0.784 | 0.769 |
| PAR × PRD | 0.194 | 0.439 | 0.491 | 0.737 | 0.304 | 0.145 |
| TRT × PAR × PRD | 0.348 | 0.001 | 0.081 | 0.953 | 0.178 | 0.223 |
| HOUR | <0.001 | <0.001 | <0.001 | <0.001 | <0.001 | <0.001 |
| TRT × HOUR | 0.019 | 0.042 | 0.088 | <0.001 | 0.002 | <0.001 |
| PAR × HOUR | 0.951 | 0.154 | 0.194 | 0.590 | 0.553 | 0.337 |
| TRT × PAR × HOUR | 0.004 | 0.025 | 0.469 | 0.986 | 0.538 | 0.051 |
| PRD × HOUR | 0.141 | <0.001 | <0.001 | <0.001 | <0.001 | <0.001 |
| TRT × PRD × HOUR | 0.086 | 0.520 | 0.571 | 0.838 | 0.072 | 0.487 |
| PAR × PRD × HOUR | 0.941 | 0.990 | 0.999 | 1.000 | 0.083 | 0.440 |
| TRT × PAR × PRD × HOUR | 0.407 | 0.384 | 0.643 | 1.000 | 0.894 | 0.923 |
| ^1^TRT = dietary treatment; PAR = parity (first vs. multiple lactations); PRD = period; HOUR = hour of the day. | | | | | | |

| **Table S15**  Effects of dietary treatment, parity, period, and hour of the day on ingestive behavior and position *P*-values for effects of dietary treatment, period, and sampling time on ingestive behavior of doelings in the first lactation and does. | | | | | | | | |
| --- | --- | --- | --- | --- | --- | --- | --- | --- |
| PAR^1^ | Item | Source of variation^2^ | | | | | | |
|  |  | TRT | PRD | TRT × PRD | HOUR | TRT × HOUR | PRD × HOUR | TRT × PRD × HOUR |
| 1 | Rumination time, min/h | 0.284 | 0.159 | 0.064 | <0.001 | 0.004 | 0.161 | 0.529 |
|  | Eating time, min/h | 0.337 | 0.009 | 0.001 | <0.001 | 0.073 | <0.001 | 0.743 |
| 2 | Rumination time, min/h | 0.134 | 0.666 | 0.689 | <0.001 | 0.102 | 0.929 | 0.034 |
|  | Eating time, min/h | 0.445 | 0.058 | 0.185 | <0.001 | 0.015 | <0.001 | 0.205 |
| ^1^Parities (PAR) were first vs. multiple lactations (1 and 2, respectively). | | | | | | | | |

^2^TRT = dietary treatment; PRD = period; HOUR = hour of the day.

**Fig. S1.** Effects of period and hour on standing time of Alpine doelings in their first lactation and does fed diets with various levels and sources of forages. An arrow showing at the x-axis indicates the feed delivery in the Calan gate feeders. Lines 1, 2, 3, and 4 indicate periods 1, 2, 3, and 4, respectively.

**Fig. S2.** Effects of period and hour on time lying on the right side of Alpine doelings in their first lactation and does fed diets with various levels and sources of forages. An arrow showing at the x-axis indicates the feed delivery in the Calan gate feeders. Lines 1, 2, 3, and 4 indicate periods 1, 2, 3, and 4, respectively.

**Fig. S3.** Effects of period and hour on time lying on the left side of Alpine doelings in their first lactation and does fed diets with various levels and sources of forages. An arrow showing at the x-axis indicates the feed delivery in the Calan gate feeders. Lines 1, 2, 3, and 4 indicate periods 1, 2, 3, and 4, respectively.

**Fig. S4.** Effects of period and hour on idle time of Alpine doelings in their first lactation and does fed diets with various levels and sources of forages. An arrow showing at the x-axis indicates the feed delivery in the Calan gate feeders. Lines 2 and 4 indicate periods 2 and 4, respectively.

**Fig. S5.** Effects of period and hour on eating time of Alpine doelings in the first lactation fed diets with various levels and sources of forages. An arrow showing at the x-axis indicates the feed delivery in the Calan gate feeders. Lines 2 and 4 indicate periods 2 and 4, respectively.

A

B

**Fig. S6.** Effects of dietary treatment and hour on ruminating time during (A) period 2 and (B) period 4 of Alpine does fed diets with various levels and sources of forages. An arrow showing at the x-axis indicates the feed delivery in the Calan gate feeders. Diets were 40%, 50%, 60%, and 70% forage (40F, 50F, 60F, and 70F, respectively), with forage in 60F and 70F diet being grass hay (primarily orchardgrass) and that in 40F and 50F cottonseed hulls, dehydrated alfalfa pellets, and wheat hay.

**Fig. S7.** Effects of period and hour on eating time of Alpine does fed diets with various levels and sources of forages. An arrow showing at the x-axis indicates the feed delivery in the Calan gate feeders. Lines 2 and 4 indicate periods 2 and 4, respectively.

**Fig. S8.** Effects of dietary treatment and hour on standing time of Alpine doelings in their first lactation and does fed diets with various levels and sources of forages. An arrow showing at the x-axis indicates the feed delivery in the Calan gate feeders. Diets were 40%, 50%, 60%, and 70% forage (40F, 50F, 60F, and 70F, respectively), with forage in 60F and 70F diet being grass hay (primarily orchardgrass) and that in 40F and 50F cottonseed hulls, dehydrated alfalfa pellets, and wheat hay.

**Fig. S9.** Effects of dietary treatment and hour on time lying on the right side time of Alpine doelings in their first lactation and does fed diets with various levels and sources of forages. An arrow showing at the x-axis indicates the feed delivery in the Calan gate feeders. Diets were 40%, 50%, 60%, and 70% forage (40F, 50F, 60F, and 70F, respectively), with forage in 60F and 70F diet being grass hay (primarily orchardgrass) and that in 40F and 50F cottonseed hulls, dehydrated alfalfa pellets, and wheat hay.

**Fig. S10.** Effects of dietary treatment and hour on time lying on the left side time of Alpine doelings in their first lactation and does fed diets with various levels and sources of forages. An arrow showing at the x-axis indicates the feed delivery in the Calan gate feeders. Diets were 40%, 50%, 60%, and 70% forage (40F, 50F, 60F, and 70F, respectively), with forage in 60F and 70F diet being grass hay (primarily orchardgrass) and that in 40F and 50F cottonseed hulls, dehydrated alfalfa pellets, and wheat hay.
